# Genome-wide identification and characterization of soybean GH9 endo-1,4-β-glucanases

**DOI:** 10.3389/fpls.2025.1597668

**Published:** 2025-06-13

**Authors:** Weimin Zhan, Huijuan Huang, Zaipan Cai, Zhenwei Zhao, Xuefu Lin, Yize Peng, Zhicheng Dong, Di Qin, Li Jiang

**Affiliations:** ^1^ Guangzhou Key Laboratory of Crop Gene Editing, Guangdong Provincial Key Laboratory of Plant Adaptation and Molecular Design, Innovative Center of Molecular Genetics and Evolution, School of Life Sciences, Guangzhou University, Guangzhou, China; ^2^ Institute of Medical Plant Physiology and Ecology, School of Pharmaceutical Sciences, Guangzhou University of Chinese Medicine, Guangzhou, China

**Keywords:** soybean, abscission cellulase, haplotype analysis, glycosyl hydrolase 9, ethylene response

## Abstract

Cellulases are a crucial class of enzymes involved in cellulose synthesis and metabolism, significantly contributing to plant growth, development, and organ abscission. The role of Glycosyl hydrolase family 9 (GH9), a major gene family encoding cellulase, remains poorly elucidated in soybean. In this experiment, we identified 43 non-redundant *GmGH9* genes in soybean through systematic genome-wide analysis. The physicochemical properties of GmGH9 proteins exhibit variability. Phylogenetic investigations revealed that class B constitutes the predominant evolutionary branch. The *GmGH9B/C* members display complex splicing patterns. GmGH9As contain typical transmembrane structural domains, while GmGH9Cs uniquely includes the carbohydrate-binding module 49 (CBM49) and signal peptide. Furthermore, we identified 13 distinct types of functional motifs, with light-responsive elements being predominant. Expression profiling of the *GmGH9s* in soybean revealed spatiotemporal and stress-regulated dynamics across organs, ethylene treatments, and photoperiodic conditions, especially for *GmGH9A9* and *GmGH9B19*. Multi-species collinearity analysis of *GH9* genes suggested that *GmGH9A2* and *GmGH9C4* exhibited greater conservation in pea, tomato, and soybean, which are distinguished by fruit abscission. Additional correlations between the haplotypes of *GmGH9A2* and *GmGH9C4* and yield-related traits indicated that soybean experienced selected pressure during domestication, resulting in a reduction in their genetic diversity.

## 1 Introduction

Plant organ abscission is a genetically organized process mediated by the coordinated activity of hydrolytic enzymes and phytohormones, which enable the specific destruction of cell wall constituents at defined abscission zone (AZ). This intricate process requires the concerted activity of various enzymes and signaling molecules, including cellulases, hemicellulases, pectinases (both polygalacturonases and pectinesterases), xylanases, expansins, peroxidases, and lipoxygenases. Notably, cellulases, which constitute one of the three biggest categories of industrial enzymes—catalyze the hydrolysis of β−1,4‐glycosidic bonds in cellulose, consequently compromising the mechanical integrity of the separation layer (Tranbarger et al., 2017).

Cellulase family comprises multiple members that can be categorized into three principal groups based on their mode of action: endo−β−1,4−glucanases (EGases), exo−β−1,4−glucanases, and β−glucosidases. The principal plant cellulases investigated to date are conventional β−1,4−glucanases, which belong to the glycosyl hydrolase family 9 (GH9). This multigene family is further divided into three distinct structural subclasses: A, B, and C. Subclass A encompasses membrane‐anchored isoforms (with or without a cytosolic domain), subclass B comprises secreted isoforms and subclass C includes secreted isoforms that contain a carbohydrate−binding module (CBM) (Urbanowicz et al., 2007). Sequence homology analyses have identified 25 *GH9* gene family members in rice (*Oryza sativa*), *Arabidopsis*, and *Populus* (Libertini et al., 2004; Xie et al., 2013), and 24, 32, 42, and 74 *GH9* genes in tomato, citrus, tobacco, and wheat, respectively (Du et al., 2015; Luo et al., 2022a; Deng et al., 2024).

The GH9 family is extensively documented for its essential functions in various biological processes, including plant organ abscission, fruit maturation, stress adaption, and developmental regulation (Robert et al., 2005; Flors et al., 2007; Lin et al., 2023; Meng et al., 2024). Members of this gene family generally display tissue-specific expression patterns influenced by environmental cues and hormone signaling. In tomato (*Solanum lycopersicum*), six members (*SlCel1-6*) of the GH9 family have been characterized, with *SlCel5* exhibiting ethylene-responsive upregulation in AZ while maintaining constant expression in stem tissues (del Campillo and Bennett, 1996). Notably, the synergistic interaction between expansin SlExp1 and endoglucanase SlCel2 mediates cell wall disassembly during fruit softening (Su et al., 2024). Three members of the *GH9A* subfamily have been parsed in *Spathiphyllum*, *SpGH9A1*, *SpGH9A2*, and *SpGH9A3*, among which the differential expression of the *SpGH9A3* gene in the leaves of *Spathiphyllum* may affect the cellulase activity, and consequently, the cellulose content of the leaves at different stages of expansion, which ultimately leads to the differences in leaf morphology (Yang et al., 2024a).

*GH9s*, express in plant pedicels, fruit stalks, and petioles, are regulated by a variety of hormones and light signals, and these regulatory mechanisms are key components of *GH9* genes involved in plant organ abscission, as well as environmental adaptation. In litchi (*Litchi chinensis*), the transcription factor LcHB2 directly activates *GH9* paralogs *LcCEL2* and *LcCEL8* within the fruit AZ, triggering sequential cellulase activation, cell wall degradation, and ultimately the organ abscission process (Li et al., 2019). *Arabidopsis* research reveals that *AtCEL6* regulates silique dehiscence by coordinating cell differentiation timing in the valve and separation layer (He et al., 2018), while *ATGH9A1/KORRIGAN1* participates in both cellulose biosynthesis and cellular elongation processes (Lane et al., 2001). The *VaGH9A1* and *VaGH1B* were expressed in the AZ of blueberry fruit (*Vaccinium ashei*) and decreased as the fruit matured, whereas the expression of *VaGH10B* and *VaGH10c* continued to increase, which is thought to be a sign that the former is involved in the pre-formation stage of cell layer differentiation in the AZ and the latter is involved in the segregation stage where cell wall breakdown is active (Wang et al., 2023c). Additionally, phytohormonal regulation of cellulase activity has been demonstrated in bean (*Phaseolus vulgaris*), where ethylene and auxin signaling pathways converge to modulate *BAC* gene expression (Tucker et al., 2002). Recent evidence suggests that in tomato, *SlIDL6* signaling may orchestrate pedicel abscission through transcriptional activation of cell wall hydrolases including TAPG1, TAPG4, and CEL2 (Li et al., 2021). SlGH9–15 has been identified as a key factor in the process of fruit cracking in tomatoes, which has been demonstrated to be activated by various hormones, such as ethylene and abscisic acid (ABA), as well as by abiotic stresses (Lin et al., 2023). Wheat *GH9* gene family, which are rich in hormone-responsive and light-responsive elements, mediate the interaction between the jasmonic acid and ABA pathways to govern another development, with seven members identified as key regulators of cellulose levels via light and phytohormones, crucial for pollen fertility and anther dehiscence (Luo et al., 2022b).

Soybean (*Glycine max*) is an important source of vegetable fats and proteins. However, the higher flower and pod abscission rates limit soybean yields, and the process is strongly influenced by the environment, including temperature, light intensity, disease, water stress, and nutrient supply, among others (Estornell et al., 2013), while the GH9 family members are involved in those processes. Suppression of soybean *GmCel7*, a soybean homologue of *AtGH9B2*, showed increased resistance to soybean cyst nematode (*Heterodera glycines*) in soybean root (Woo et al., 2014). A hard-seed allele *qHS1* from *Glycine soja* (*G. soja*) was identified as *GmGH9B8* for increasing the amount of β-1,4-glucans in the outer layer of palisade cells of the seed (Jang et al., 2015). Using *qHS1* loci for improvement of the modern cultivar “Tachinagaha” making its seed coat less permeable and more resistant to cracking. Similar to tomato, increased cellulase activity in the soybean AZ of pedicels and petioles may suggest that the *GH9s* family is also involved in the progress of organ abscission in soybean (Kemmerer and Tucker, 1994; Tucker et al., 2007). However, there are few reports about the roles of *GH9s* participating in the regulation of organic development in soybeans.

With the construction of the soybean pan-genome, the attainment of comprehensiveness, diversity, and in-depth functional resolution for soybean gene research is achieved (Liu et al., 2020). This study presents a comprehensive analysis of the chromosomal localization, phylogenetic relationships, gene structure, promoter regulatory motifs, and tissue expression profiles of the soybean cellulase gene family, characterized by the GH9 structural domain, at the genome-wide scale. We predicted the *GmGH9s* that are specifically expressed and responsive to ethylene hormone signaling in the soybean AZ to further explore the potential GH9 family members involved in soybean organ abscission and ultimately constructed the haplotypes of *GmGH9A2* and *GmGH9C4*. Our study establishes a theoretical foundation for further understanding comprehension of *GmGH9s*’ function and offers a new perspective for investigating the mechanism of soybean organ abscission.

## 2 Materials and methods

### 2.1 *GmGH9s* identification and physicochemical properties analysis

*Glycine max* (Williams 82, Wm82) reference genome (Wm82 V6), along with its genomic sequences, protein annotations, and structural features in GFF3 format, was retrieved from SoyBase (Grant et al., 2010). Members of the GH9 family candidate were identified through a dual screening strategy. Homology-based screening: blastp alignments (E-value cutoff <1e-5) against *Arabidopsis* GH9 family members. Domain validation: Hidden Markov Model (HMM) profiling with the PF00759 seed alignment (Pfam v35.0) (Mistry et al., 2021) via HOMER v5.1 (Eddy, 2011), followed by SMART (Letunic et al., 2021) and CDD v3.21 (Wang et al., 2023b) database analyses to confirm GH9 specific catalytic domains (cd00254, glycosyl hydrolase family 9). Biochemical characteristics of GmGH9 proteins, such as molecular weight (MW), theoretical isoelectric point (pI), and grand average of hydropathicity (GRAVY), were computed using ExPASy ProtParam (Gasteiger et al., 2003) with default settings.

### 2.2 Chromosome localization and collinearity analysis of GmGH9s

Genomic coordinates of validated *GmGH9* genes were extracted from the GFF3 file and visualized using TBtools-II v2.156 (Chen et al., 2023) with the “Advanced Chromosome Map” module. Whole-genome tandem duplication events were detected through MCScanX v1.0.0 (Wang et al., 2024) with default parameters (blastp E-value <1e-10, match score >50). Genomic and GFF3 files for pea, tomato, *Arabidopsis*, and maize were downloaded from the Ensembl plant database (release 60) (Bolser et al., 2016). Twelve soybean pangenomes (SoyW02, SoyW03, SoyL02, SoyL03, SoyC02, SoyL05, ZH13, SoyC11, SoyC12, SoyL09, and SoyC14) and their corresponding GFF3 files were downloaded from the SoyMD database (Yang et al., 2024b). Segmental duplication analysis through synteny blocks identified using JCVI v1.5.1 utilities (Tang et al., 2024). Ka/Ks ratios were calculated via KaKs_Calculator 3.0 (Zhang, 2022) to characterize evolutionary selection pressure.

### 2.3 Phylogenetic tree analysis

A multiple sequence alignment of soybean and other model plants (*Arabidopsis* [dicotyledonous] and maize [monocotyledonous]) was performed using the Clustalw algorithm in MEGA X (Kumar et al., 2018). The evolution analysis was performed using the maximum likelihood with default parameters, and a bootstrap of 1000 was applied. Finally, the evolutionary tree was visualized on the Evolview3 (Subramanian et al., 2019) webserver, with GH9s labeled accordingly.

### 2.4 Gene structure and conserved motif analysis

Exon-intron structures of *GmGH9* genes were extracted from the GFF3 file, and gene structure schematics were visualized using TBtools-II v2.156. Conserved protein motifs were identified using the MEME Suite v5.5.2 (Bailey et al., 2006) with parameters set to a maximum of 4 motifs, E-value<1e−5, and motif widths ranging from 6 to 50 residues. The distribution of conserved motifs was mapped onto the phylogenetic tree using TBtools-II v2.156 to elucidate structural conservation across the *GmGH9* family.

### 2.5 Promoter and *cis*-regulatory element analysis

Promoter sequences (2000 bp upstream of the transcription start site) of *GmGH9* genes were retrieved from the soybean genome (Wm82.v6). *Cis*-regulatory elements were predicted using PlantCARE v2023 (Lescot et al., 2002) and categorized into functional groups (e.g., light-responsive, hormone-related, and stress-inducible elements). The distribution of these elements was visualized in R v4.3.1 using the ggplot2 package, with color-coded annotations highlighting regulatory diversity among subfamilies.

### 2.6 Haplotype analysis

SNP data of 4414 soybean accessions were obtained from the SoyMD database (Yang et al., 2024b). Yield trait data were obtained from the SoyOmics database (Liu et al., 2023). Haplotype blocks for *GmGH9* genes were analyzed using geneHapR (Zhang et al., 2023), with haplotypes containing fewer than 10 individuals being excluded from the analysis. With a 20 kb window and 2 kb step, VCFtools v0.1.16 (Danecek et al., 2011) was used to calculate the nucleotide diversity (π) and fixation index (F*st*) values. The top 10% of the π and F*st* ratio (*G. soja vs*. landrace and landrace *vs*. cultivar) for the corresponding chromosome was used as the selective sweep threshold.

### 2.7 Material planting

Soybean variety Wm82 was selected and cultivated in a greenhouse. The growth conditions included a photoperiod of 16 hours (h) light/8 h dark, maintained at a temperature of 26°C. Fifteen days after flowering, soybean plants received a treatment of 100 mg/L ethephon, with water treatment serving as control. After 24 h, tissue samples were designated as ethylene-treated stem (ESt), ethylene-treated leaf (EL), ethylene-treated petiole (EYb), ethylene-treated flower (EF), ethylene-treated flower AZ (EFaz), ethylene-treated pod AZ (EPaz), ethylene-treated pod peel (EPp), ethylene-treated seed coat (ESc), and ethylene-treated embryo (ESe). Parallel samples were harvested from water-treated plants as control, designated as stem (St), leaf (L), petiole (Yb), flower (F), flower AZ (Faz), pod AZ (Paz), pod peel (Pp), seed coat (Sc), and embryo (Se). All samples were frozen in liquid nitrogen and stored at -80°C.

### 2.8 Analysis of the expression profile and RT-qPCR

Data on multi-organ expression (NCBI BioProject ID: PRJNA442256), AZ and NAZ *in situ* expression following ethylene treatment (Kim et al., 2016), and expression data from photoperiod conversion [plants were grown in the greenhouse under short-day (SD) (10 hours of light, 6:45–16:45) and long-day (LD) (16 hours of light, 6:45–22:45) conditions at 25°C and were sampled every four hours at six time points, T1-T6 (6:30, 10:30, 14:30, 18:30, 22:30 and 2:30). For a shift (Sh) experiment, plants were first grown under LD for three weeks and then transferred to SD for 5 days] (Wu et al., 2014) were downloaded from the publicly accessible transcriptome dataset in the NCBI database. Raw data was filtered using Trimmomatic software (Bolger et al., 2014), followed by alignment of clean data to the reference genome Wm82 v6 using STAR (Dobin et al., 2013). The counts of all genes were ultimately converted to gene expression values in transcripts per million (TPM).

RNA was isolated via the TRIzol method (Rio et al., 2010), and the concentration and integrity were evaluated with a NanoDrop spectrophotometer. RNA integrity was detected by 1.2% agarose gel electrophoresis. cDNA was synthesized using HiScript III RT SuperMix (Vazyme International LLC, Nanjing, China). Primers for real-time fluorescence quantitative PCR (RT-qPCR) were designed by Primer-BLAST online (Ye et al., 2012), and listed in **Supplementary Table S6**. RT-qPCR was performed using SYBR Green qPCR Mix (Thermo Fisher Scientific, Waltham, USA), with 40 cycles set. *GmTublin* was set as the internal reference gene, and gene expression level was calculated using the 2^(-ΔΔCt [cycle threshold]) method (Livak and Schmittgen, 2001).

### 2.9 Subcellular localization

*GmGH*-GFP (green fluorescent protein) fused expression cassettes were inserted into the pCAMBIA1302 vector with restriction sites (*Hin*dIII and *Kpn*I), subsequently transformed into *GV3101*(pSoup-P19) *Agrobacterium* strain, and used to infect tobacco (*Nicotiana benthamiana*) leaves via injection. GFP fluorescence signals were detected after 48 h using a confocal laser scanning microscope (Zeiss LSM 880). AtPIP2A, connected to mCherry as a plasma membrane localization control (Li et al., 2024). The primer sequences used in this study are presented in **Supplementary Table S6**.

### 2.10 Data analysis

Heatmaps of expression data were generated in R v4.3.1 using the pheatmap package, with Z-score normalization. Statistical significance (*p*<0.05) was assessed via Student’s *t*-test to compare expression differences across tissues or treatments.

## 3 Results

### 3.1 Identification analysis of *GmGH9s* in *Glycine max*

Through systematic genome-wide analysis using HMMER3.0, we identified 43 non-redundant *GmGH9* genes in soybeans, with strict elimination of redundant isoforms. Bioinformatic analysis revealed substantial variation in the physicochemical properties of encoded GH9 proteins. The polypeptides ranged from 126 amino acids (aa) (GmGH9B14) to 639 aa (GmGH9C4), corresponding to molecular weights between 14.62 kDa (GmGH9B14) and 70.76 kDa (GmGH9A16) (**Supplementary Table S1**). Theoretical isoelectric points (pI) exhibited broad diversity from 4.46 (GmGH9A6) to 9.52 (GmGH9B1), while the grand average of hydropathy (GRAVY) values spanned from -0.432 (GmGH9A16) to 0.291 (GmGH9A4). Notably, only two proteins (GmGH9A4 and GmGH9B18) displayed positive GRAVY scores, indicating that 95.3% of GmGH9 proteins are hydrophilic. These distinct physicochemical characteristics suggest functional diversification of GmGH9 proteins across cellular environments.

### 3.2 Phylogenetic analysis and classification of GmGH9s

**Figure 1:**
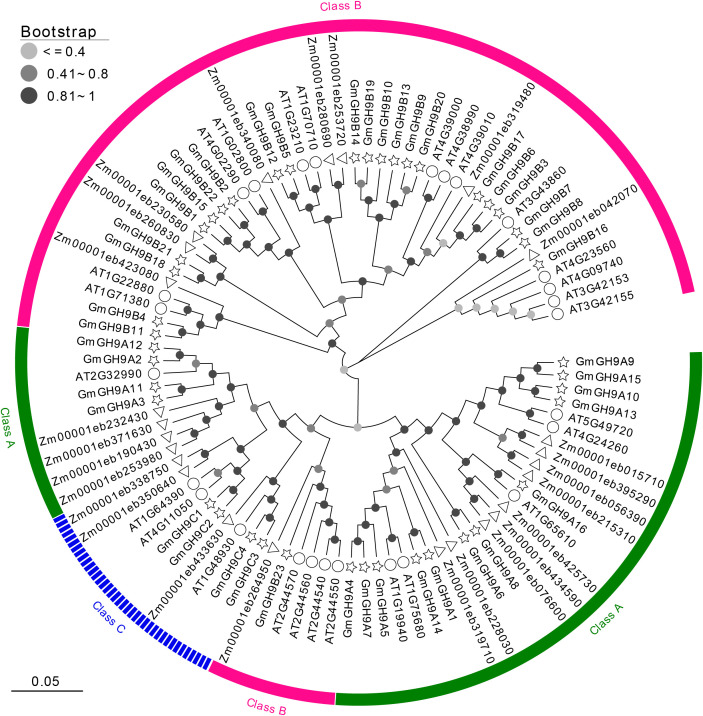
Phylogenetic tree of multi-species GH9s. Green, pink, and blue represent Class A, Class B, and Class C subgroups, respectively. Different leaf decorations represent different species. The color of the circle on the branch represents different bootstrap values.

To elucidate the evolutionary phylogenomics of GH9s glycosyl hydrolases, we performed maximum-likelihood phylogenetic reconstruction using MEGA X with 1000 bootstrap (BS) replicates, stratifying nodal support into three confidence tiers: low (BS ≤ 0.4), moderate (0.41<BS ≤ 0.8), and high (BS>0.8), with topological robustness quantified in strongly supported nodes (BS≥0.81) (**Figure 1**; **Supplementary Table S1**). The phylogram incorporated cross-kingdom homologs from Arabidopsis (*Arabidopsis thaliana*) and maize (*Zea mays*), revealing deep phylogenetic conservation of cellulose-metabolizing GH9 enzymes across embryophytes. According to the classification standards for *Arabidopsis* GH9s (Urbanowicz et al., 2007), the 43 GmGH9 predicted proteins segregated into three evolutionarily distinct classes: Class A (n=16, 37.21%), Class B (n=23, 53.49%), and Class C (n=4, 9.30%), demonstrating phyletic divergence consistent with subfunctionalization patterns. Class B emerged as the predominant clade, exhibiting functional diversification with varying nodal support (e.g., GmGH9B16/B7/B8) in contrast to an evolutionarily constrained cluster (e.g., GmGH9B11-B4/AT1G71380/AT1G22880/Zm00001eb423080). Intriguingly, phylogenetically cohesive subgroups of tandemly duplicated soybean paralogs (e.g., GmGH9A12/A2/A11/A3) formed species-specific expansions, potentially reflecting neofunctionalization events following whole-genome duplication.

### 3.3 Chromosomal location and collinearity analysis of *GmGH9s*

**Figure 2:**
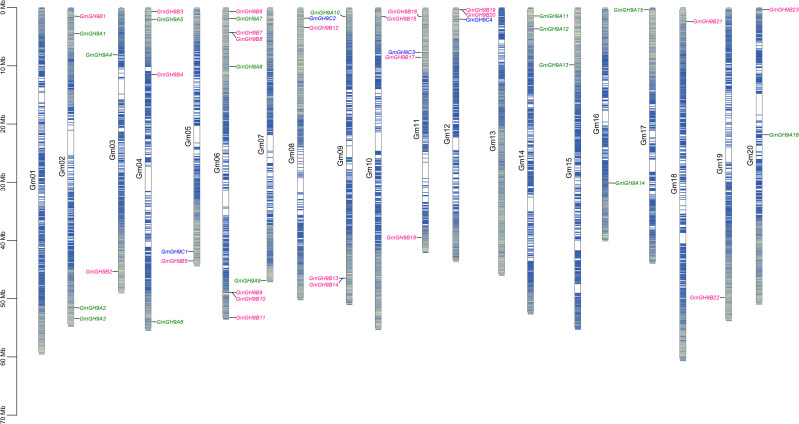
Chromosome distribution of *GmGH9s*. Green, pink, and blue represent Class A, Class B, and Class C subgroups, respectively.

The 43 *GmGH9s* exhibited a non-random chromosomal distribution across 18 of the 20 soybean pseudomolecules, notably absent from Gm01 and Gm13 (**Figure 2**). Chromosomal allocation analysis revealed Gm06 as a genomic hotspot, harboring 23.3% (8/43) of *GmGH9* members, followed by Gm02, Gm04, and Gm11, each with 4 genes, representing 9.3% per chromosome. Eight Class B members (GmGH9B7/B8, B9/B10, B13/B14, B19/B20) particularly formed tandem arrays within 200 kb intervals, predominantly localized in pericentromeric regions—a genomic architecture suggestive of non-reciprocal transpositions or unequal crossing-over events. A comparative analysis identified 28 segmental duplication pairs (**Supplementary Figure S1**), predominantly associated with ancient whole-genome duplication (WGD) events, as evidenced by synonymous substitution rates (Ks=0.09–2.61) and strong purifying selection (Ka/Ks=0.03–0.66; **Supplementary Table S2**). These evolutionary trajectories likely fine-tuned the spatiotemporal regulation of cell wall polysaccharide remodeling, facilitating adaptive responses to mechanical stress and pathogen threats in soybean ecotypes.

### 3.4 Conserved motifs, conserved domains, and gene structures of the GH9 family in soybean

**Figure 3:**
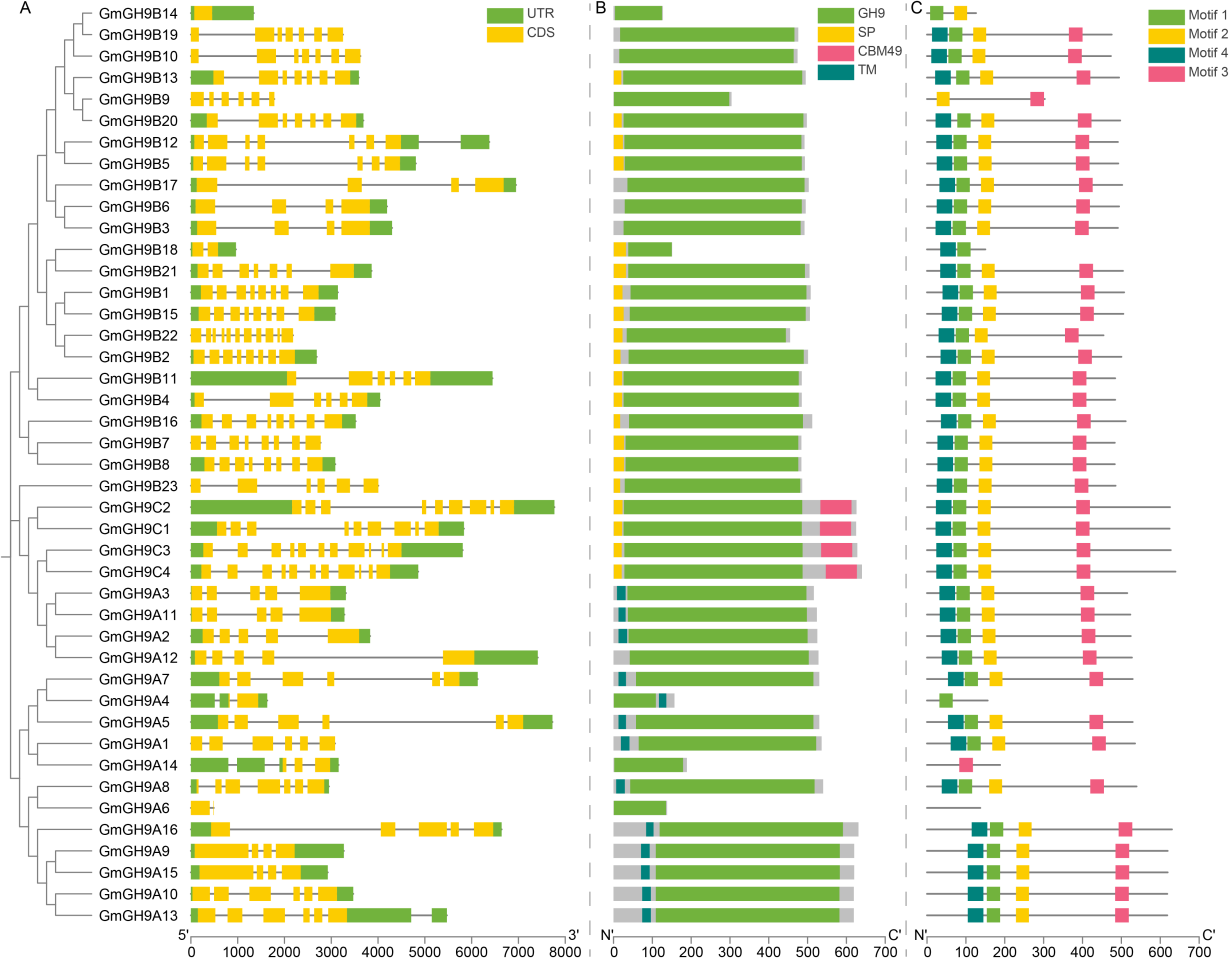
Molecular characteristics of GmGH9s. **(A)** *GmGH9* structures; grey lines represent introns. **(B)** GmGH9 proteins conserved domain; the gray area is disordered region. **(C)** GmGH9 conserved motif; gray line is relatively disordered sequence.

Comprehensive phylogeny-structural analysis of the soybean GmGH9 family unveiled conserved evolutionary modules and subfamily-specific innovations across GmGH9A/B/C clades. Exon-intron architectural complexity diverged significantly (**Figure 3A**), with GmGH9B/C members displaying elaborate splicing patterns (e.g., the 3’UTRs [three prime untranslated regions] of *GmGH9B12* and *GmGH9A13* contain introns) in contrast to the streamlined *GmGH9A* genes. These subfamily-differentiated UTR configurations, particularly intron-containing 3’UTRs, suggest *cis*-regulatory diversity via post-transcriptional regulation mediated by alternative splicing.

Domain architecture (**Figure 3B**) highlighted the canonical GH9s catalytic domain, combined with distinctive characteristics peculiar to each subfamily: transmembrane (TM) domains were prominent in GmGH9As (except GmGH9A6/A12/A14), suggesting roles in membrane anchoring. GmGH9Cs uniquely harbored carbohydrate-binding module 49 (CBM49) and signal peptides (SP), implicating involvement in cellulose recognition and secretion mechanisms. These structural patterns align with phylogenetic divergence, indicating that the complexity and extension of the GmGH9Bs domain signify neofunctionalization in cellulose metabolism, whereas the streamlined architectures of GmGH9Cs imply conserved catalytic functions. Surprisingly, GmGH9A6 does not have a TM domain, and there is only a GH9 domain in the pan-gene (**Supplementary Table S3**). Phylogenetic and domain architecture may exhibit incongruence, potentially reflecting functional divergence through domain loss during lineage-specific evolution.

Motif decomposition revealed evolutionarily constrained modularity (**Figure 3C**; **Supplementary Figure S2**): four core motifs (1-4; 34–41 aa) showed positional conservation in 86.05% (n=37) of the members. Whereas features with functional loss, such as GmGH9B14/B9/B18, contain only two of the four motifs; GmGH9A4/A14 contains only one of the four motifs. GmGH9A6 inadvertently exhibited an absent pattern. The observed divergence among subfamilies may result from the tetraploidization and subsequent diploidization processes in soybean evolution, while lineage-specific adaptations may potentially underlie the cell wall remodeling strategies and ecological resilience mechanisms that govern soybean’s environmental responsiveness.

### 3.5 Analysis of *cis*-elements in the promoters and functional annotation of *GmGH9s*

**Figure 4:**
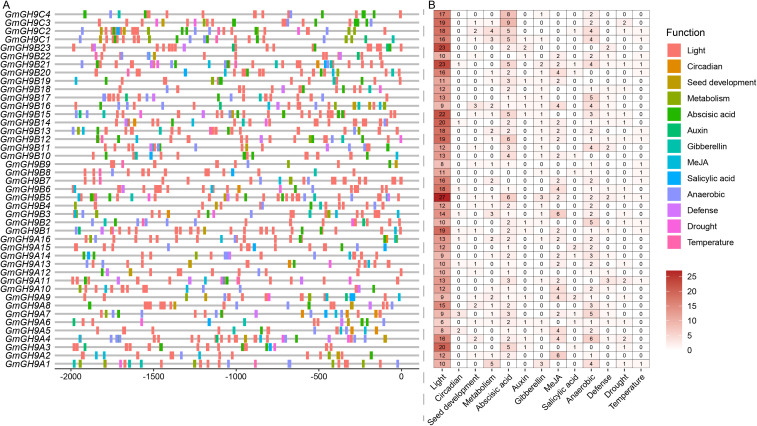
*GmGH9s* promoter motif distribution. **(A)** The distribution of different motifs in the promoter position. **(B)** The heatmap of motifs function classification statistics.

Analysis of the promoters of the soybean *GmGH9* gene family revealed a diverse array of *cis*-regulatory elements associated with biological processes and stress responses. A total of 13 types of functional motifs were identified (**Figure 4**), with all gene promoters containing light-responsive motifs, consistent with the diurnal regulation of cellulose metabolism (Wang et al., 2023a). Moreover, the distribution of light-responsive motifs is relatively dense, with *GmGH9B5*, *GmGH9B21*, and *GmGH9B23* containing 27, 23, and 23 light signal motifs, respectively. Hormone-responsive elements are notably predominant, including ABA (39/43), gibberellin (GA, 24/43), methyl jasmonate (MeJA, 24/43), auxin (13/43), and salicylic acid (SA, 11/43) signaling pathways, suggesting tight hormonal regulation of *GmGH9s* expression. Stress-related motifs were highly enriched, particularly those linked to anaerobic conditions (37/43), drought (18/43), and temperature fluctuations (15/43), underscoring the family’s potential role in abiotic stress adaptation. Seed development-related motifs were also abundant, highlighting the family’s involvement in growth processes. *GmGH9B5* exhibited a higher motif density for light and ABA metabolic regulation, whereas *GmGH9B9* contained only 13 motifs. We performed GO (**Supplementary Table S4**) and KEGG (**Supplementary Table S5**) enrichment analysis to examine the function of GmGH9s. It was found that they were mainly engaged in the biological process of “cell wall modification”, subsequently followed by “response to cyclopentenone” and “β-glucan metabolic process” (related to cytokinin) (**Supplementary Figure S3**). Pathway enrichment analysis identified starch and sucrose metabolism as the most significantly enriched KEGG pathway. These findings collectively suggest that *GmGH9* genes are regulated by a complex interplay of developmental, hormonal, and environmental cues, likely fine-tuning their roles in cell wall remodeling and stress resilience in soybeans.

### 3.6 *GmGH9s* expression profiles under different conditions

**Figure 5:**
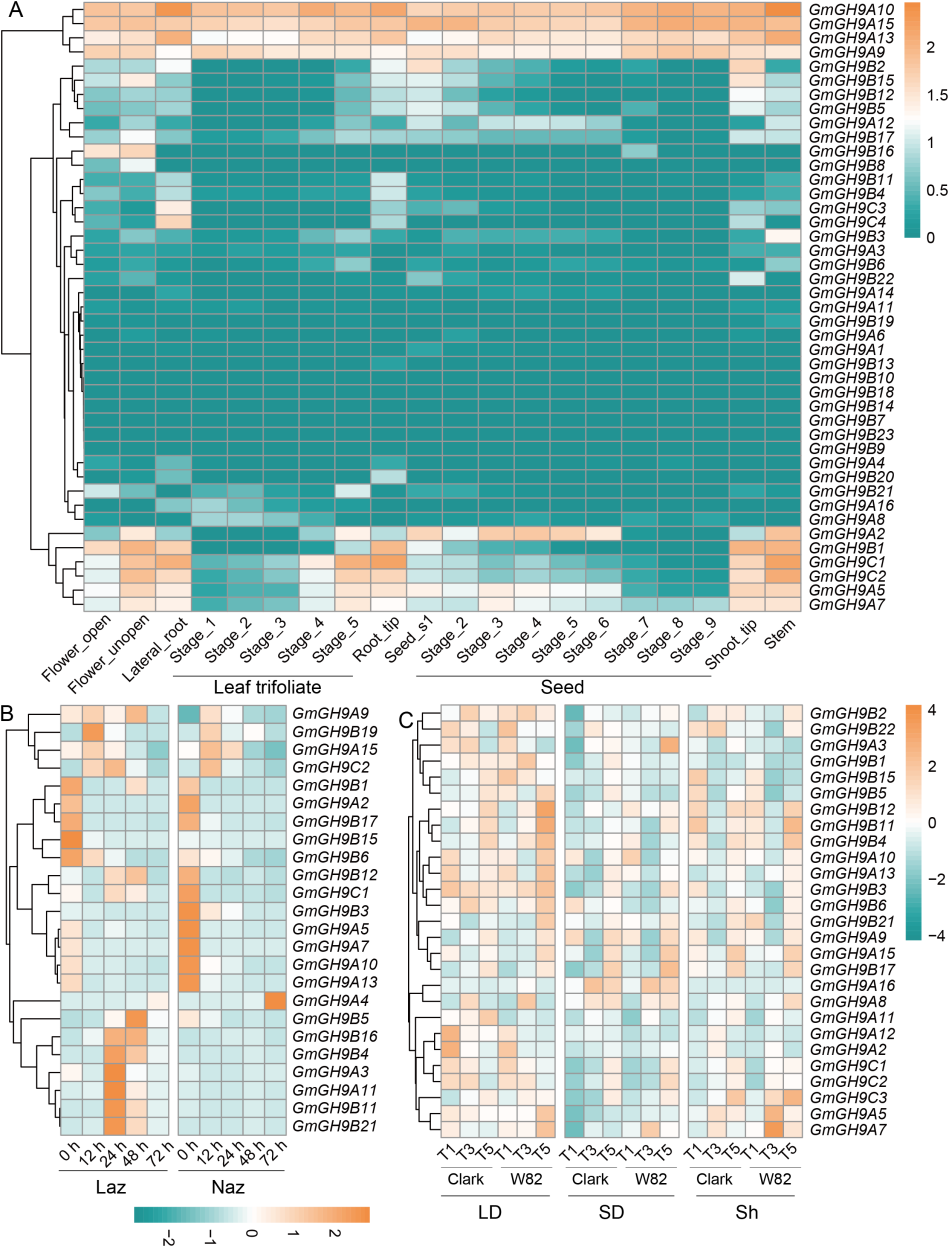
Spatio-temporal expression of *GmGH9s*. **(A)** The expression of *GmGH9s* in different organs and periods. **(B)** The expression of *GmGH9s* in leaf abscission zones (Laz) and non-leaf abscission zones (Naz) at 0 hour (h), 12 h, 24 h, 48 h and 72 h after ethylene induction. **(C)**, *GmGH9s* in soybean varieties Clark and Williams 82 (Wm82) long-day (LD, 16 h of light, 6:45-22:45) and short-day (SD, 10 h of light, 6:45-16:45) period three weeks after germination. Expression patterns at T1 (6:30), T3 (14:30), and T5 (22:30) under LD, SD, and light conversion (Sh, first grown under LD for three weeks and then transferred to SD for 5 days) conditions.

The expression profiling of the *GmGH9s* family in soybean revealed spatiotemporal and stress-responsive regulatory dynamics across organs, ethylene treatment, and photoperiod conditions (**Figures 5A–C**). Organ-specific expression highlighted functional diversification (**Figure 5A**). *GmGH9C4* was highly expressed in lateral roots, while *GmGH9C1* and *GmGH9C2* were specifically expressed at the apical regions (root and shoot). Seed-specific expression peaks of *GmGH9A2* correlated with seed maturation. In addition, *GmGH9A10/15/13/9* showed constitutive expression. Ethylene-responsive regulation (**Figure 5B**) revealed distinct expression patterns in leaf AZ (Laz) compared to non-AZ (Naz). *GmGH9B15* was highly expressed in Laz-specific at 0 h after ethylene induction (AEI). *GmGH9B19* was significantly induced by ethylene in Laz compared to Naz at 12 h AEI. Key genes (*GmGH9B21/B11/A11/A3/B4/B16/B5*) exhibited significant induction in Laz at 24–48 h AEI, consistent with ethylene’s role in cell wall loosening during abscission. In contrast, *GmGH9A4* was downregulated in Laz but upregulated in Naz at 72 h AEI, indicating compartmentalized regulatory roles. Photoperiod-dependent expression (**Figure 5C**) demonstrated that LD conditions enhanced the expression of *GmGH9B2/B22/A3/B1/B15/B5/A12/A2/C1/C2*, while SD conditions suppressed it. SD significantly inhibited gene expression, except *GmGH9A16* and *GmGH9A8*. Strikingly, *GmGH9B12*, *GmGH9B11*, and *GmGH9B4* displayed rhythmic oscillations under LD, with peaks occurring at dawn (T5), and demonstrated elevated expression levels at T5 in Sh. Surprisingly, most genes showed a similar expression pattern in Clark and Wm82.

**Figure 6:**
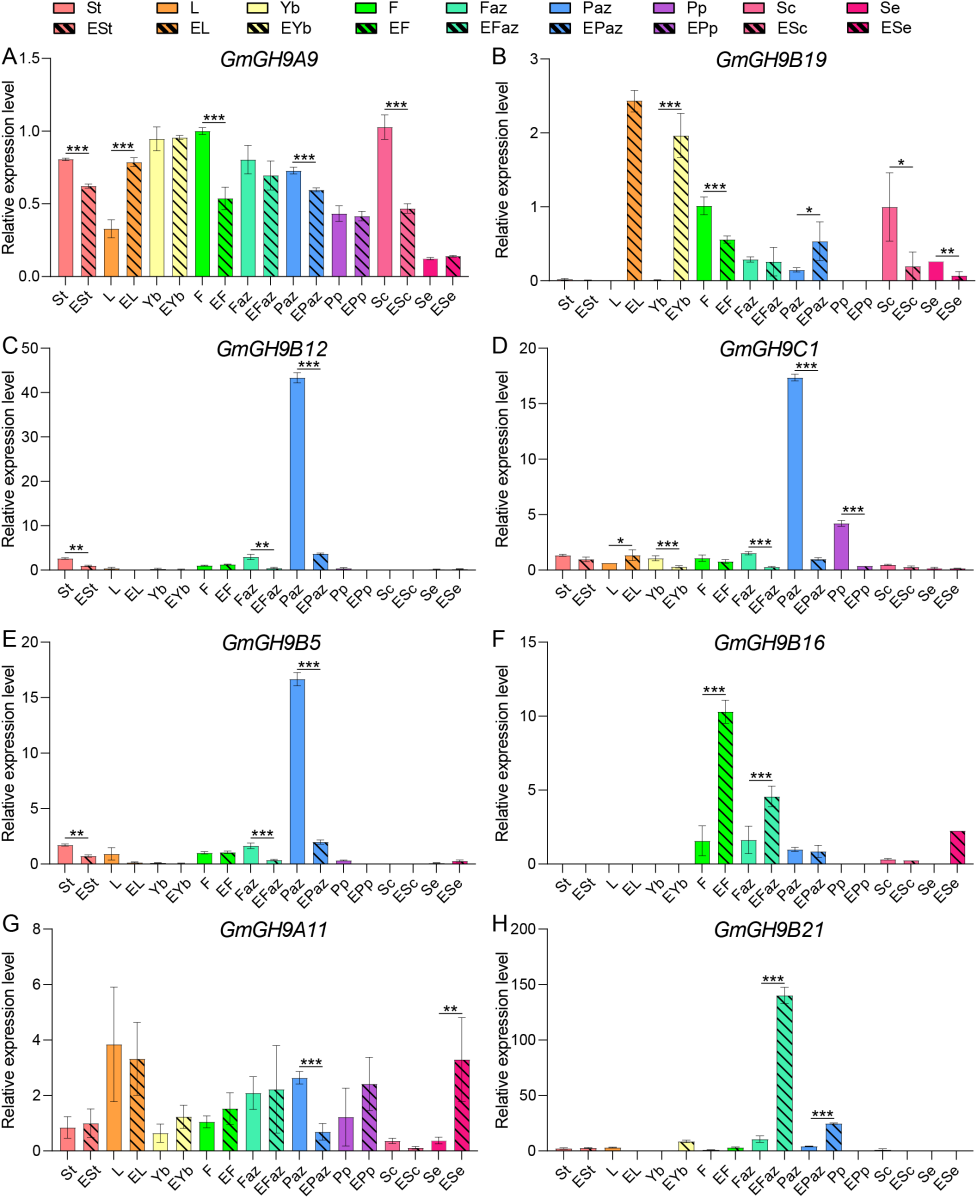
RT-qPCR analysis of the response of *GmGH9s* to ethylene in different tissues. **(A–H)** The relative expression levels of *GmGH9A9*, *GmGH9B19*, *GmGH9B12*, *GmGH9C1*, *GmGH9B5*, *GmGH9B16*, *GmGH9A11*, and *GmGH9B21* respectively. ESt, EL, EYb, EF, EFaz, EPaz, EPp, ESc, and ESe denote ethylene-treated stem, leaf, petiole, flower, flower AZ, pod AZ, pod peel, seed coat, and embryo, respectively. Data are presented as means ± SEM. *, **, and *** indicate significant differences at *p*<0.05, *p*<0.01, and *p*<0.001, respectively.

RT-qPCR was employed for examining the changes in gene expression of *GmGH9* genes, which show different patterns (either tissue-specific high expression profiles across soybean organs, or induced or suppressed by ethylene) (**Figure 5B**). Stem, leaf, petiole, flower, Faz, Paz, pod peel, seed coat, and embryo were detected before and after ethylene treatment. As shown in **Figures 6A, B**, ethylene significantly enhanced the expression of *GmGH9A9* and *GmGH9B19* in leaves, whereas concurrently repressing their expression in flowers and the seed coat. *GmGH9B12*, *GmGH9C1*, and *GmGH9B19* were found to be specifically highly expressed in the Paz. However, following ethylene treatment, their expression was dramatically decreased, showing a 15 to 35 fold reduction. In the Faz, both *GmGH9B16* and *GmGH9B21* exhibited increased expression levels in response to ethylene. Furthermore, *GmGH9B16* was notable for its up-regulation in flower, whereas *GmGH9B21* showed a significant rise in expression in the Paz, achieving a three-fold increase relative to control levels. The results indicate that *GmGH9s* may have multiple roles in abscission.

### 3.7 Multi-species colinearity analysis of *GH9s*

**Figure 7:**
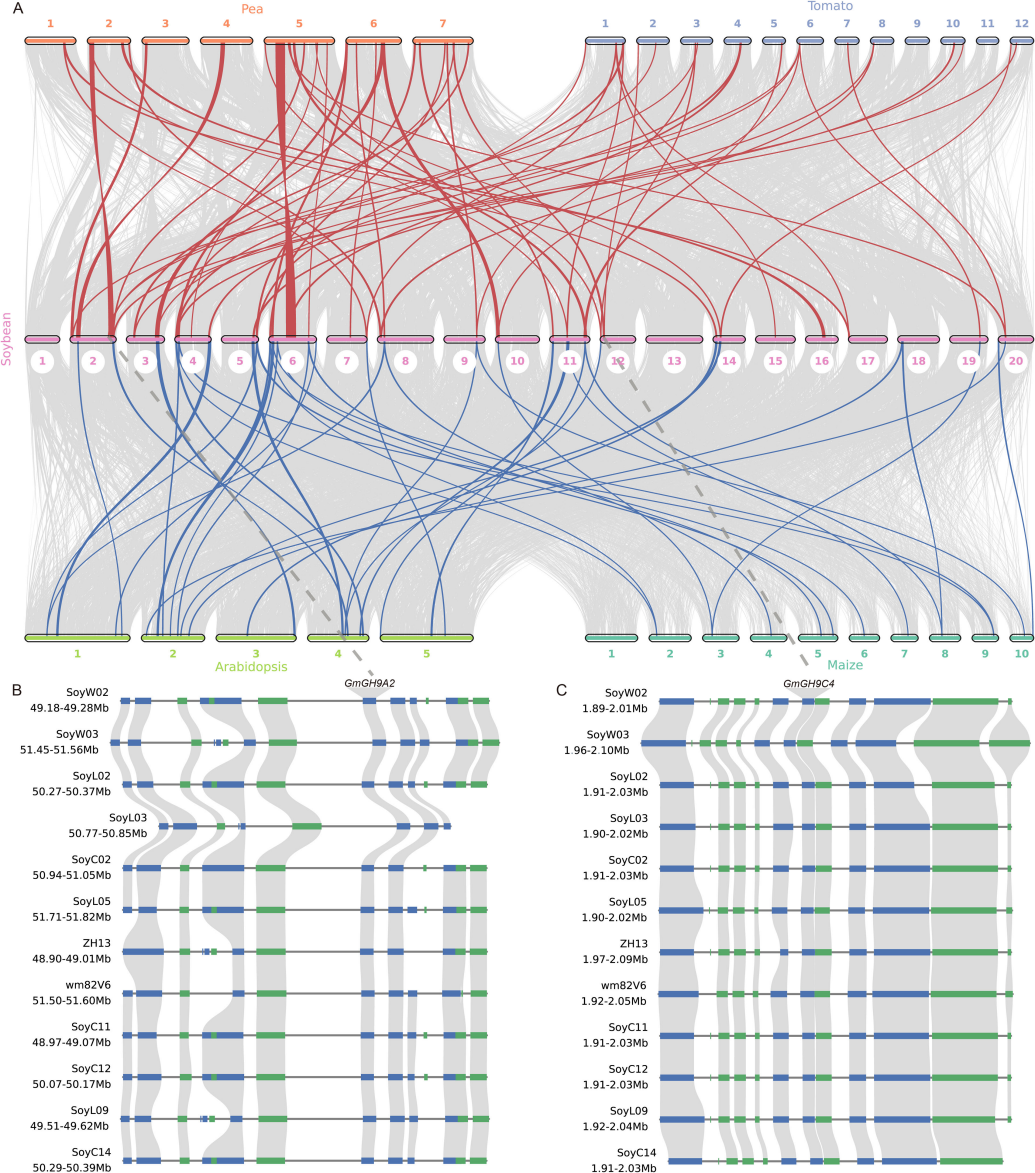
Co-linearity analysis of *GH9s*. **(A)** Multi-species genome-wide collinearity analysis. **(B)** Microsynteny analysis of the *GmGH9A2* within soybean species. **(C)** Microsynteny analysis of the *GmGH9C4* within soybean species. Green and blue represent genes on the negative and positive strands of chromosomes, respectively.

**Figure 8:**
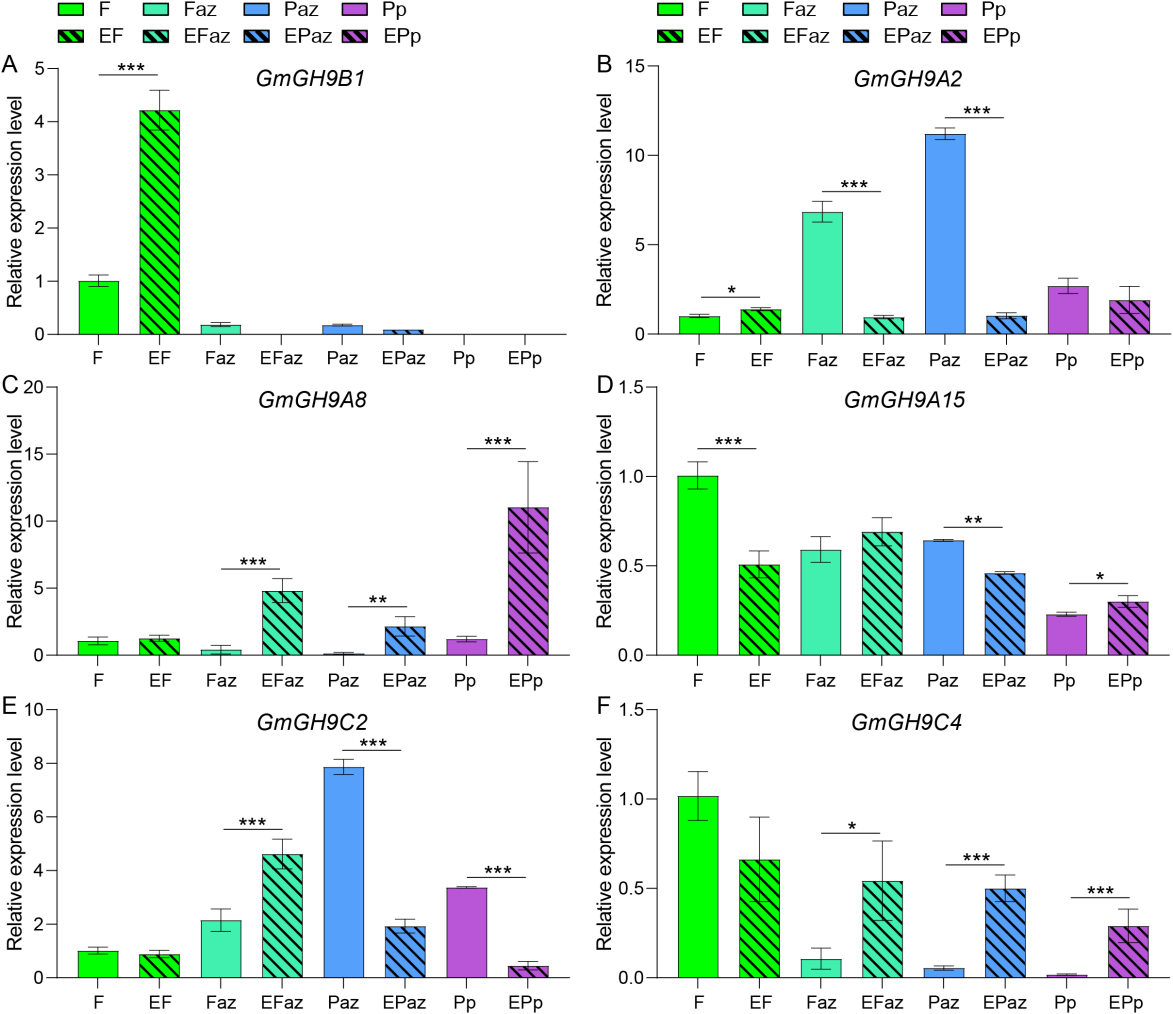
RT-qPCR analysis of the response of *GmGH9s* to ethylene in distinct floral and pod zones. **(A–F)** The relative expression levels of *GmGH9B1*, *GmGH9A2*, *GmGH9A8*, *GmGH9A15*, *GmGH9C2*, and *GmGH9C4*, respectively. Data are presented as means ± SEM. *, **, and *** indicate significant differences at *p*<0.05, *p*<0.01, and *p*<0.001, respectively.

The comparative synteny analysis of the *GmGH9s* family among soybean, pea (*Pisum sativum*), tomato (*Solanum lycopersicum*), *Arabidopsis*, and maize revealed both conserved and divergent evolutionary patterns. Colinearity analysis for the GH9s gene family revealed that pea, tomato, *Arabidopsis*, and maize exhibit 30, 25, 23, and 15 collinear events with soybean, respectively. The syntenic region shared among all species includes *GmGHB3/B5/B13/B14/B17/B18*. Moreover, seven GH9 syntenic regions—specifically *GmGH9B1/A2/A4/A8/C2/C4/A15*—are conserved in pea, tomato, and soybean, while they are absent in *Arabidopsis* and maize. These genes exhibit a high level of conservation throughout the soybean pan-genome (**Figures 7B, C**; **Supplementary Figures S4–S8**). Notably, *GmGH9B1/A4/C2/A15* displayed differential expression in leaves between Laz and Naz after ethylene treatment (**Figure 5B**). *GmGH9B1* exhibited specific expression in flowers following ethylene induction, with negligible levels detected in other organs (**Figure 8A**). *GmGH9A2* showed a significant reduction in the AZ of flowers and pods after ethylene treatment (**Figure 8B**). Conversely, *GmGH9A8* and *GmGH9C4* exhibited significantly increased expression in the AZ of flowers and pods following ethylene treatment, suggesting their potential involvement in the regulation of flower and pod abscission (**Figures 8C, F**). In contrast, *GmGH9C2* showed opposite ethylene responses in the AZ of flowers and pods, implying functional diversity (**Figure 8E**). Additionally, *GmGH9A15* displayed differential expression in the Paz, whereas no such expression was observed in the Faz (**Figure 8D**). Fruit or pod abscission occurs during the growth of pea, tomato, and soybean. It is hypothesized that *GmGH9A2/A8/A15/C2/C4* are linked to flower pod abscission, with *GmGH9A8/C4* facilitating this process and *GmGH9A2* serving as an inhibitor.

### 3.8 Haplotype variations of *GmGH9A2* and *GmGH9C4*

**Figure 9:**
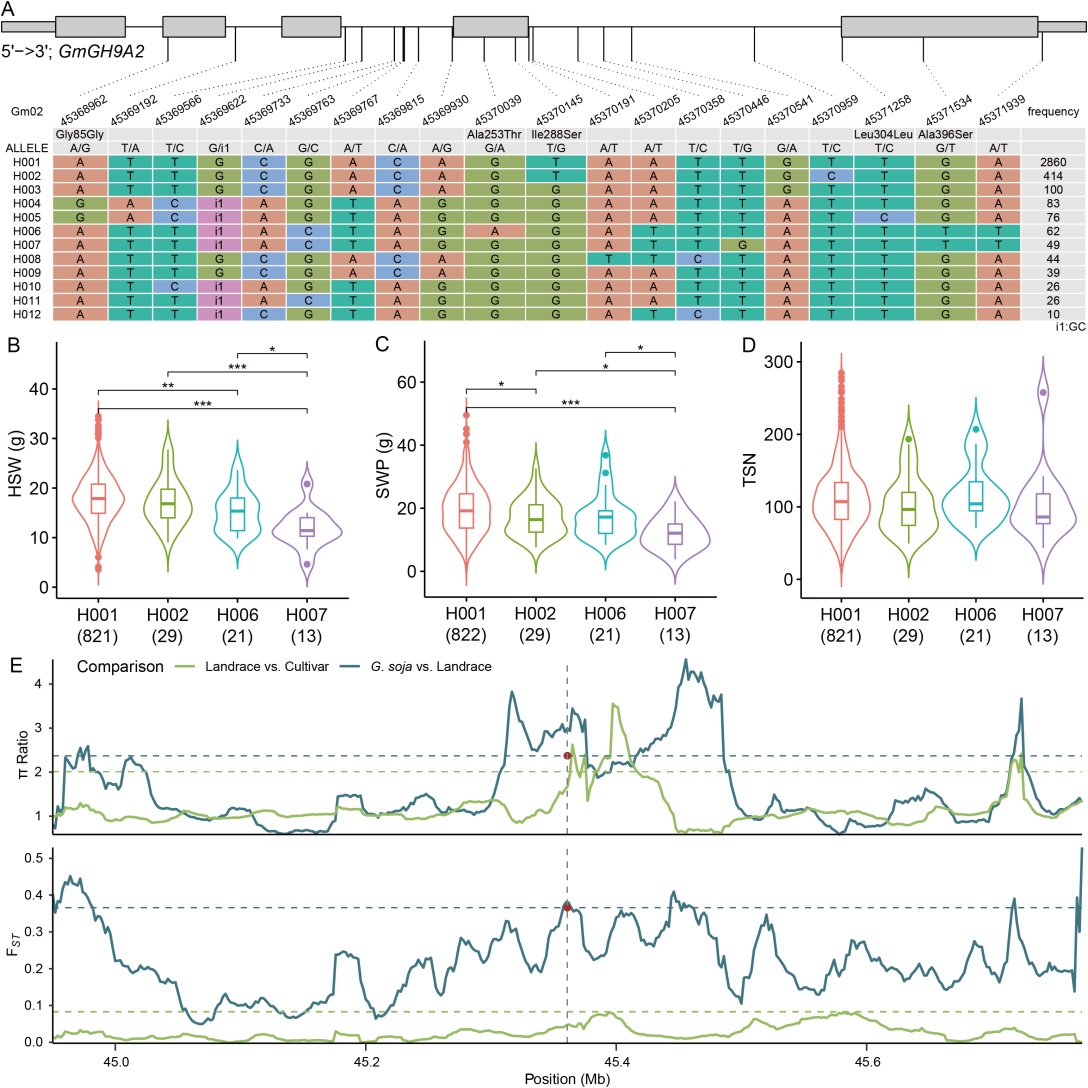
Analysis of genetic characteristics of *GmGH9A2*. **(A)** Haplotype of *GmGH9A2*. The wide and narrow gray boxes represent the exon and UTR regions, respectively. The gray line represents the intron. **(B–D)** The relationship between haplotypes with 100-seed weight (HSW), seed weight per plant (SWP), and total seed number (TSN). **(E)** The π ratio and *F*st values of the flanking region at *GmGH9A2* in (*G*) *soja*, landraces, and cultivars soybeans. The horizontal dashed lines indicate the genome-wide thresholds of (*G*) *soja vs*. landraces, and landraces *vs*. cultivars (top 10%). The middle position of *GmGH9A2* is labeled by brown dot (Gm02: 45370242). *, **, and *** indicate significant differences at *p*<0.05, *p*<0.01, and *p*<0.001, respectively.

To clarify the effects of *GmGH9s* on soybean morphology, we examined the association between the haplotypes of *GmGH9A2* and *GmGH9C4* with yield-related traits, including 100‐seed weight (HSW), seed weight per plant (SWP), and total seed number per plant (TSN). *GmGH9A2* and *GmGH9C4* had twelve and five haplotypes, respectively (**Figure 9A**; **Supplementary Figure S9A**). Despite the absence of differences in TSN among the haplotypes of *GmGH9A2*, H001 exhibited a significantly higher HSW compared to others, resulting in a notable improvement in SWP for H001 (**Figures 9B–D**). An analysis of π and F*st* in the 400 kb upstream and downstream regions of *GmGH9A2* among *G. soja*, landrace, and cultivated soybeans revealed that the π ratio and F*st* value for *G. soja*/landrace comparisons reached the top 10% threshold, while the F*st* between landraces and cultivars was significantly lower than that observed between *G. soja* and landraces. The π ratio of landrace/cultivar, though not exceeding the threshold, was greater than 1. This suggests that H001 was selected for enhanced yield traits in soybean cultivation and indicates that intensive breeding practices have further eroded genetic variation in cultivated lines (**Figure 9**). In the case of *GmGH9C4*, although a slight decrease in the TSN of H003, the HSW was higher than that of H001 and H002, leading to a significant improvement in SWP (**Supplementary Figures S9B–D**). In contrast to *GmGH9A2*, the π ratio of landrace/cultivar approached 1, whereas the π ratio of *G. soja*/landrace exceeded that of landrace/cultivar. This suggests that selection pressure was exerted on *G. soja* during domestication, with only modest selection changes occurring during subsequent breeding improvement (**Supplementary Figure S9E**). These findings highlight the impact of domestication and breeding on the narrowing genetic diversity at functionally critical *GmGH9A2* and *GmGH9C4*, potentially limiting adaptive potential in modern cultivars.

### 3.9 Subcellular localization of GmGH9s protein

**Figure 10:**
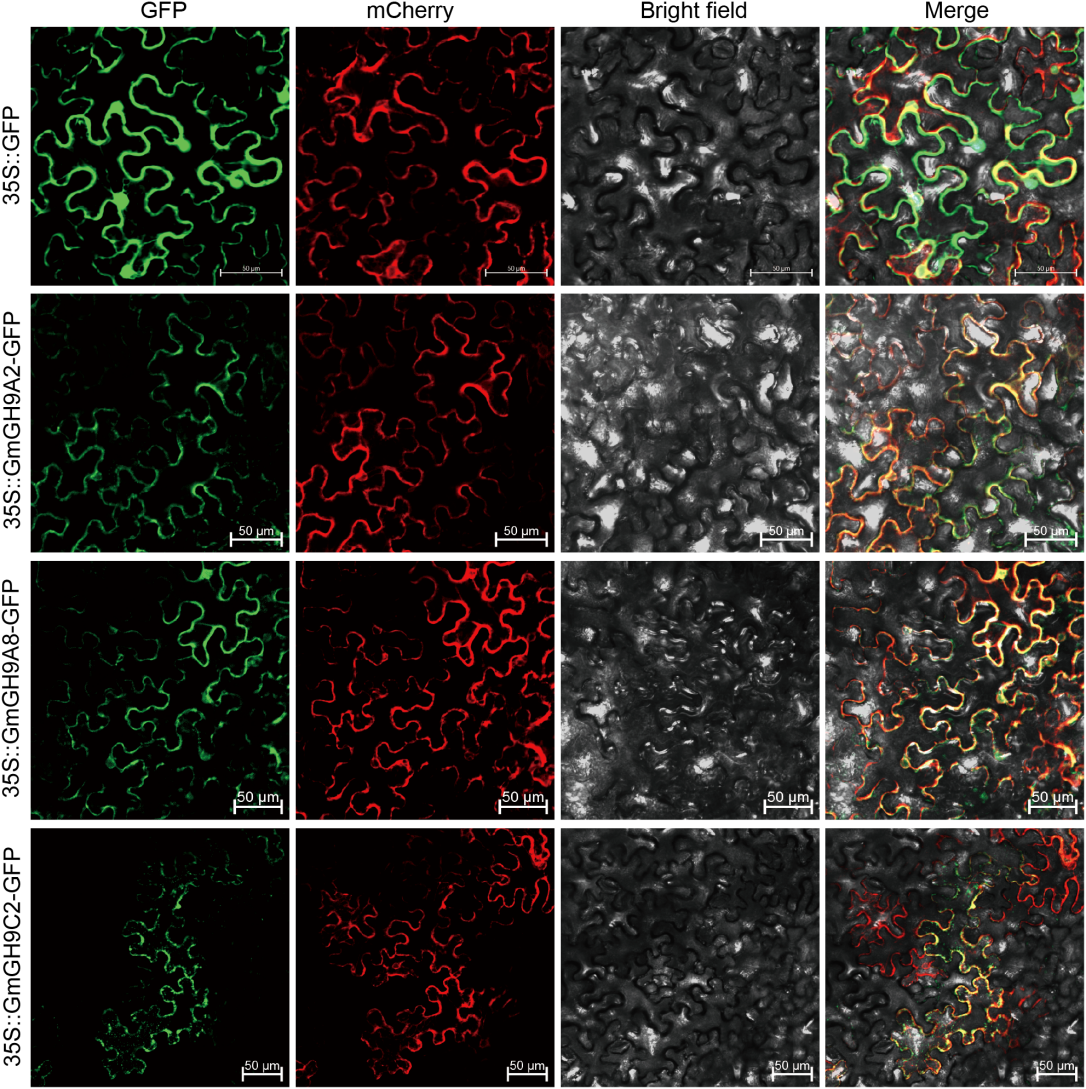
Subcellular localization of GmGH9s. GmGH9A2-GFP, GmGH9A8-GFP, and GmGH9C2-GFP fusion proteins, as well as GFP alone, were transiently expressed in *Nicotiana benthamiana* leave cells. An mCherry-based AtPIP2A labeling plasma membrane. Representative photos of the protein localization analysis conducted 48 h after infiltration are displayed. Individual images of GFP, mCherry, or bright field autofluorescence are shown. GFP merged with mCherry as well as bright field (Merge) images are also displayed. Scale bars=50 μm.

To analyze the subcellular localization of GmGH9s, we generated recombinant constructs encoding 35S::GmGH9A2-GFP, 35S::GmGH9A8-GFP, and 35S::GmGH9C2-GFP. These were co-bombarded with the plasma membrane marker 35S::AtPIP2A-mCherry into *Nicotiana benthamiana* epidermal cells via transient expression. Confocal laser scanning microscopy revealed distinct localization patterns: while the 35S::GFP control exhibited diffuse cytoplasmic localization, all three GmGH9 fusion proteins showed pronounced extracellular compartmentalization (**Figure 10**). This extracellular localization profile aligns with their predicted roles in apoplastic processes involving cell wall remodeling.

## 4 Discussion

Previous studies have documented the role of GH9s in the reassembly and degradation of the soybean cell wall (Nawaz et al., 2017). However, a comprehensive analysis of the expansion of the GH9 gene family and the subsequent functional differentiation within this family has not been conducted to date. This study performed a genome-wide identification and characterization of cellulase genes, facilitating the understanding of the genetic mechanisms underlying soybean organ abscission.

### 4.1 Gene family expansion and functional differentiation

*GmGH9* family shows considerable expansion within the soybean genome, an ancient tetraploid specie, with a notable prevalence in the GmGH9B subfamily, comprising 23 out of 43 members, paralleling observations in other species (Luo et al., 2022b; Wang et al., 2023c). This expansion is likely related to the functional diversification of cellulose metabolism. The Class C family is quite conserved, particularly in the unique CBM49 structural domain (**Figure 3B**) and the transmembrane domains of GmGH9C. This suggests a distinct role in substrate recognition and membrane localization. All members present in the multispecies covariance belong to the Class C family (**Figure 1**). The GmGH9A subfamilies are more streamlined in structure and may retain conserved catalytic functions (**Figures 3A, B**). Intriguingly, while GmGH9A3/A11/A2/A12/B23 phylogenetically clusters near class C members, it notably lacks the characteristic CBM49 domain observed in this group. This structural divergence suggests a complex evolutionary relationship between subgroup A and B with C, though their precise phylogenetic origins require further molecular evidence to resolve.

Importantly, subfunctionalization or neofunctionalization after gene duplication can occur via structural domain reorganization, such as variations in motif arrangement (Sandve et al., 2018), thereby facilitating adaptation to the dynamic remodeling of the soybean cell wall, including pectin degradation and responses to environmental stressors. Tandem repeats and segmental repeat events on chromosomes may primarily driving this expansion, with a large number of tandem repeats in the soybean genome (Zhang et al., 2024). We identified 8 tandem repeats and 28 fragment repeats (**Figure 2**; **Supplementary Figure S1**). The structural hallmarks—tandem clusters in recombinationally active zones and retained paleoduplicates—imply compartmentalized functional diversification. This may occur through subfunctionalization that preserving ancestral cellulose synthase enhancement activity or neofunctionalization that introduces novel β-1,4-glucanase specificities, potentially driven by subgenome dominance during post-polyploid diploidization.

### 4.2 Adaptive significance of *cis*-regulatory elements

Homeostasis regulation is closely related to gene expression, with alteration in cis-regulatory sequences contributing to the diversification of gene functions in soybeans (Fang et al., 2023). Promoter analysis revealed a wide range of hormone- and adversity-responsive elements (e.g., ABA, MeJA, drought, and light-responsive elements) within the *GmGH9s* gene family, suggesting that its expression is regulated by complex environmental and developmental signals. The late-stage softening and ripening of fruit are closely related to light (Li et al., 2021; Lin et al., 2023; Wei et al., 2023; Su et al., 2024). The enrichment of light signals in the *GmGH9s* promoter suggests an important role for these genes in the late stage of soybean pod development (**Figure 4**). Enrichment of elements related to photoperiod and biological clock, such as *GmGH9B5*, *GmGH9B21*, and *GmGH9B23*, further supports a role for this family in circadian-regulated cellulose metabolism. Photoperiod-dependent circadian oscillations, such as the rhythmic expression of *GmGH9A2* and *GmGH9A12* under long day light (**Figure 5B**), may be associated with carbon allocation strategies. These observed patterns are highly compatible with *cis*-elements in promoters, indicating a synergistic optimization of regulatory and functional modules during evolution.

### 4.3 Spatio-temporal specificity of expression patterns

Expression profiling revealed that *GmGH9* genes were highly specific in organ development, including root, flower, stem, and seed development, as well as in stress response such as ethylene-induced AZ formation. The high expression of *GmGH9C1* in root tips may correlate with its involvement in root cell wall expansion, whereas the up-regulation of *GmGH9A2* in late seed development (**Figure 5A**). Simultaneously, *GmGH9A2* was also rapidly expressed in Laz and Naz after ethylene treatment; conversely, its expression was suppressed in the AZ of flowers and pods (**Figures 5B**, **8B**). It reflects the functional diversity of GmGH9A2 in the petiole, flower and pod AZ. Low expression levels of certain genes were detected in different organs; however, these genes may have outstanding contributions at different times. Different organs of *GmGH9A9* and *GmGH9B19* have different sensitive responses to ethylene (**Figures 6A, B**), indicating that they have diverse functions. Despite *GmGH9B4* displaying low expression levels in various organs, the expression of Laz was significantly higher than that of Naz following 24 h of ethylene treatment (**Figure 5B**). At the same time, *GmGH9B4* also showed periodic expression, with high levels observed during the T5 stage of LD (**Figure 5C**). The up-regulation of *GH9B3* (GmGH9B4 in this study) gene expression during homograft adhesion was consistently observed in soybeans, while it was not evident in interfamily grafts (Notaguchi et al., 2020). This space-time specific expression could enhance the practicality of soybeans.

### 4.4 Implications of covariance and evolutionary history

Syntenic blocks were identified between soybean chromosomes and homologous regions in other species, highlighting ancestral genomic conservation. Collinearity analyses showed that some members of the GmGH9 family, such as the GmGH9B subfamily, share conserved syntenic blocks (**Figure 7A**), which supports their derivation from a common ancestral core gene. This “conserved core-dynamic fringe” evolutionary model may have balanced functional stability with adaptive innovation. However, the absence of synteny in monocotyledons, such as *Arabidopsis* and maize, reflects lineage-specific adaptation. Abscission of pea, tomato and soybean fruits/pods occurs during the ripening stage (Ayeh et al., 2009; Kim et al., 2016; Lin et al., 2023). The presence of *GmGH9B1/A2/A4/A8/C2/C4/A15* in pea, tomato and soybean suggests a potential relationship between these genes and fruit abscission. Overall, the synteny network underscores the dual evolutionary trajectory of the GmGH9 family: core functions are conserved through syntenic orthologs, while species-specific innovations driven by genomic plasticity.

### 4.5 Decay of genetic diversity and breeding challenges

The hydrolysis of plant cell walls leads to a series of reactions, such as HSW, a loss-of-function mutant allele of a glycosyl hydrolase gene, utilized to regulate seed weight during soybean domestication (Wei et al., 2023). *qHS1*, an endo-1,4-β-glucanase gene, harbors a SNP mutant that changes the permeability of soybean seed coat (Jang et al., 2015). Organ abscission can severely affect yield, with haplotypes of *GmGH9A2 and GmGH9C4* showing a correlation to yield (**Figure 8C**; **Supplementary Figure S9C**). Genetic diversity analyses of *GmGH9A2* revealed a significantly high π ratio and F*st* between *G. soja* and landrace (**Figure 9E**), suggesting that artificial selection has led to a genetic bottleneck at this locus. This phenomenon may arise from directional selection for specific agronomic traits in breeding, such as cell wall strength or resistance to downy mildew. The attenuation of genetic diversity from *G. soja* soybean to cultivars serves as a caution that over-reliance on a limited genetic base may weaken the capacity of future varieties to adapt climate change (Tian et al., 2025). Therefore, the use of local germplasm or the restoration of key allelic variants through gene editing may provide new strategies to optimize GmGH9s function while balancing high yield and stress tolerance. Although GmGH9s are correlated with yield, further experimental evidence is required to determine whether abscission is the causative factor.

## 5 Conclusions

In this study, 43 *GH9* genes were identified in soybean. These genes were classified into three groups. Notably, several genes exhibited significant responses to ethylene treatment and circadian variations, especially *GmGH9A9* and *GmGH9B19*. Furthermore, multi-species synteny analysis indicated that *GmGH9A2* and *GmGH9C4* may be involved in organ abscission and significantly affect soybean yield, providing valuable insights for future soybean molecular breeding. Its evolutionary history and changes in genetic diversity offer important insights for crop improvement.

## Data Availability

The original contributions presented in the study are publicly available. This data can be found here: NCBI BioProject, accession PRJNA442256 and PRJNA219510.
