# Supplementary material for: Genome-wide identification and characterization of soybean GH9 endo-1,4-β-glucanases
Source: Front Plant Sci. 2025 Jun 13;16:1597668. doi: 10.3389/fpls.2025.1597668 (PMC12202608; doi:10.3389/fpls.2025.1597668)
Supplement: Supplementary file 1 [file Supplementaryfile1.docx]

Supplementary Material


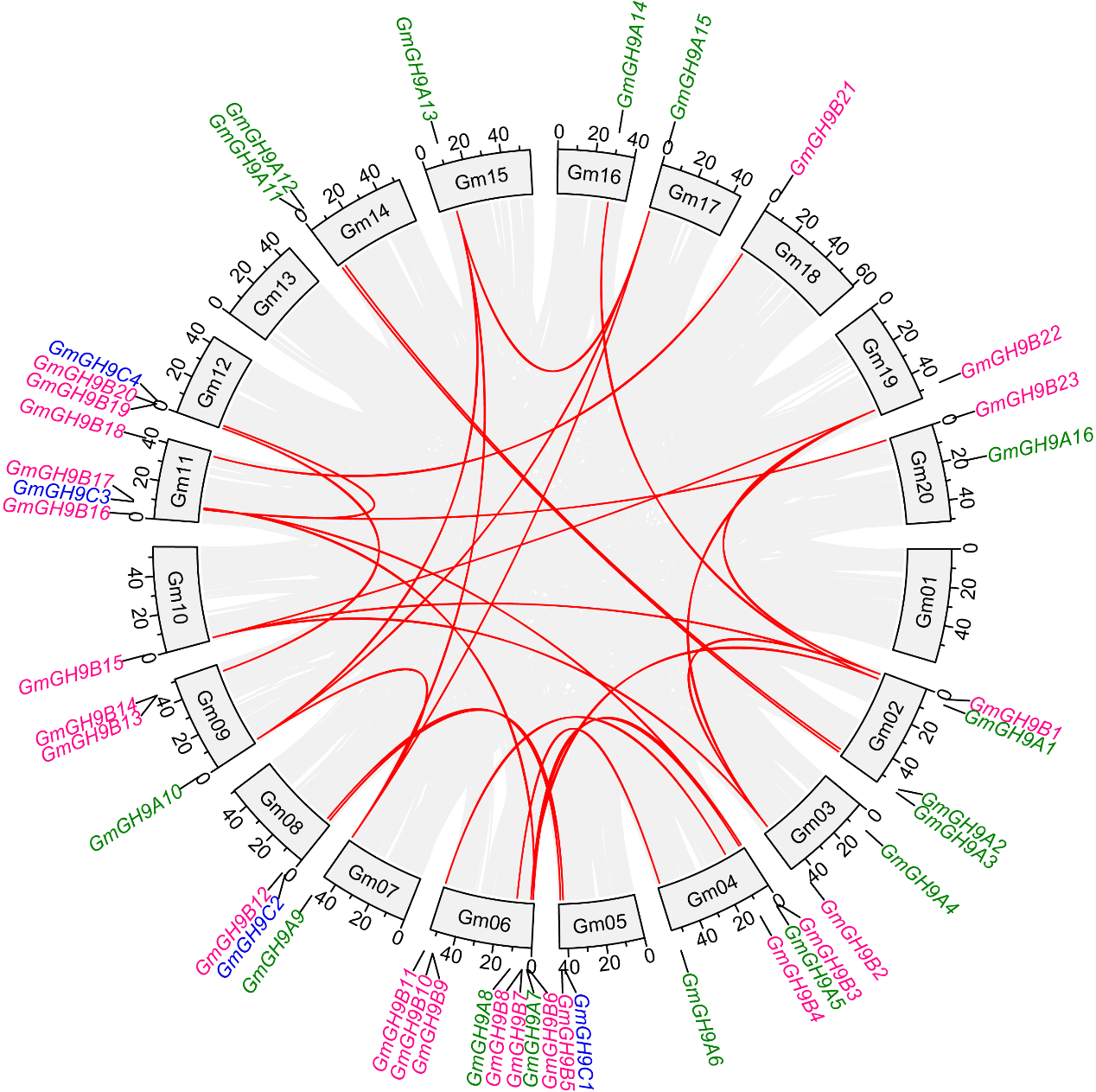


**Figure 1. *GmGH9s* gene replication events.** Green, pink and blue represent Class A, Class B and Class C subgroup, respectively. The gray lines represent genome-wide collinearity events, and the red lines represent the collinearity events of GmGH9s.


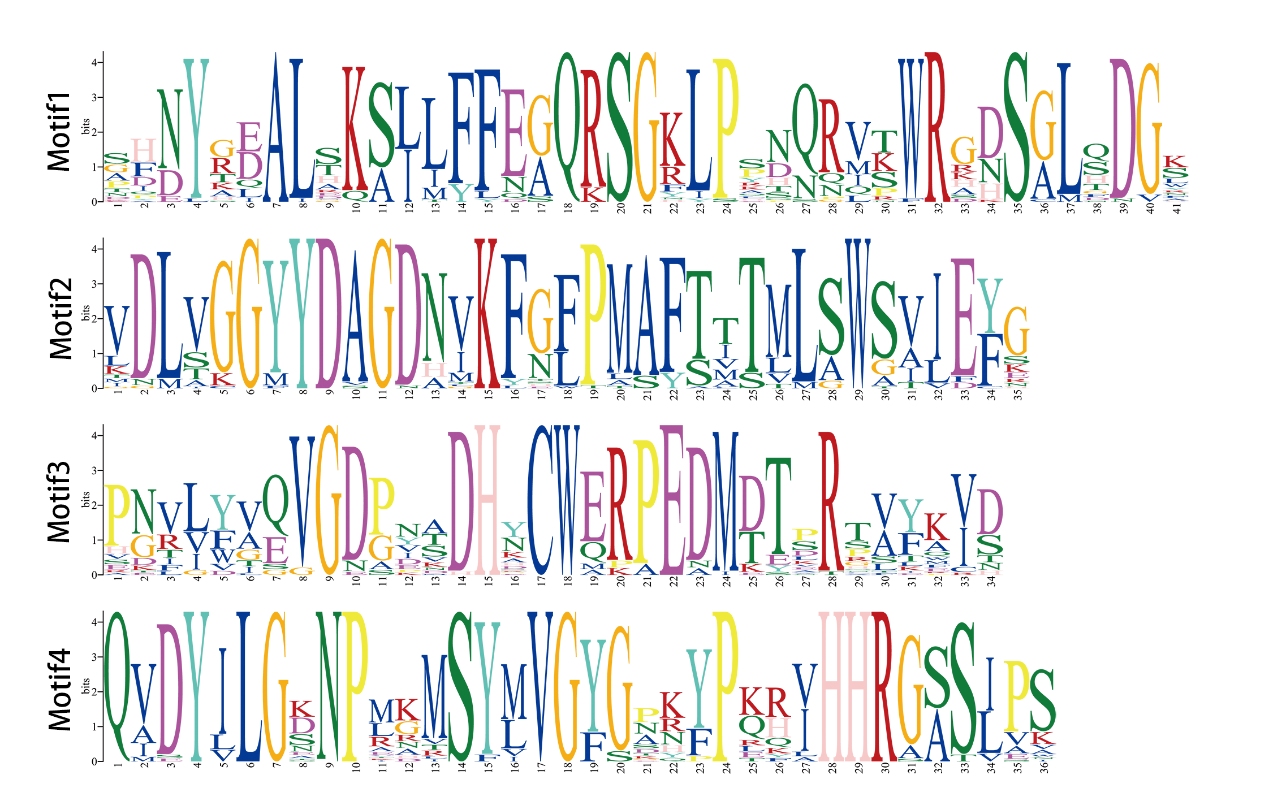


**Figure S2. Conserved motifs in GmGH9s amino acids.**


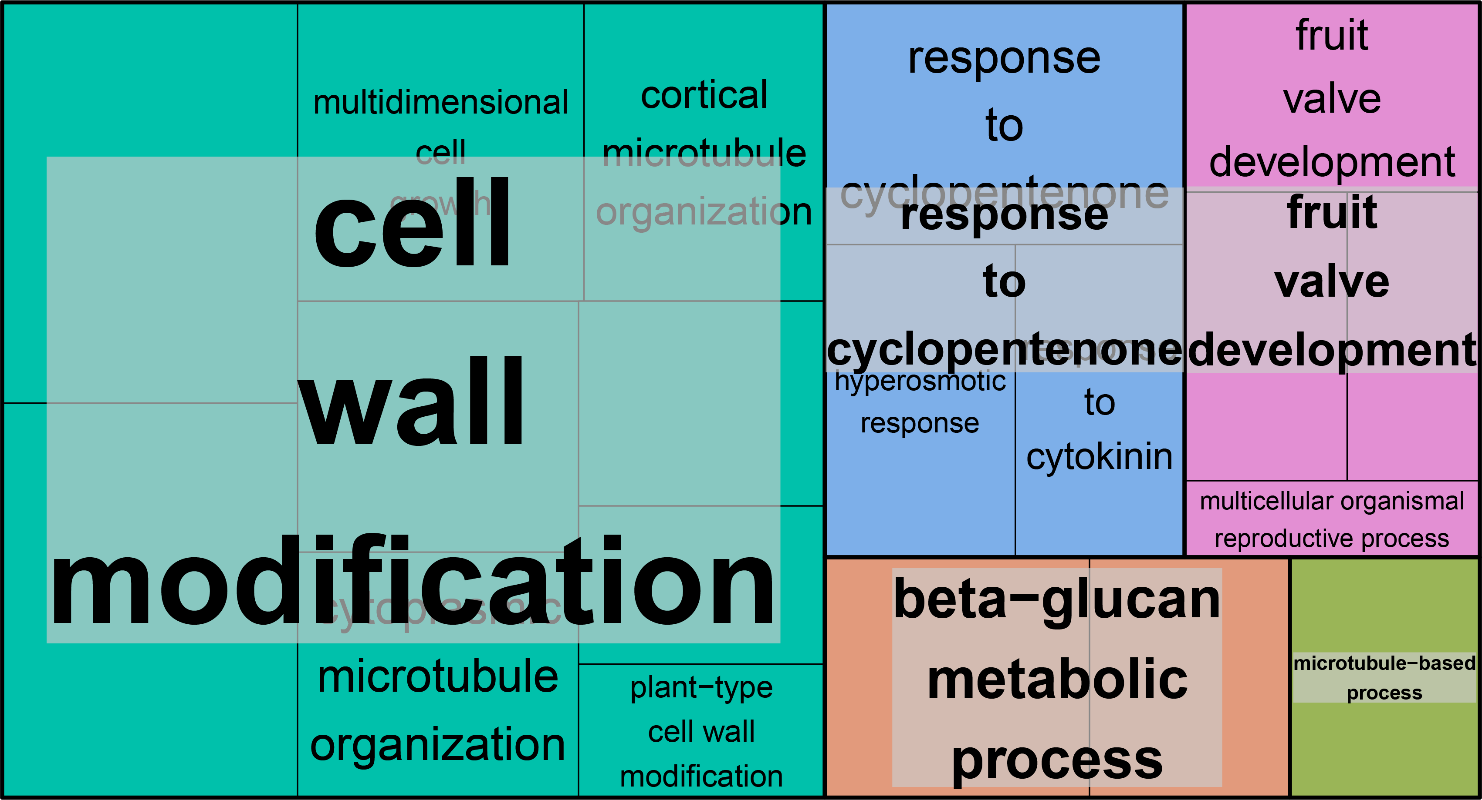


**Figure S3. GO treemap of overrepresented GO terms.** Each box represents the -log10 (*p*-value) of a single GO term, and the larger size of box reflects the most important GO term. Similar functional categories with semantic similarity are represented by similar colored boxes.


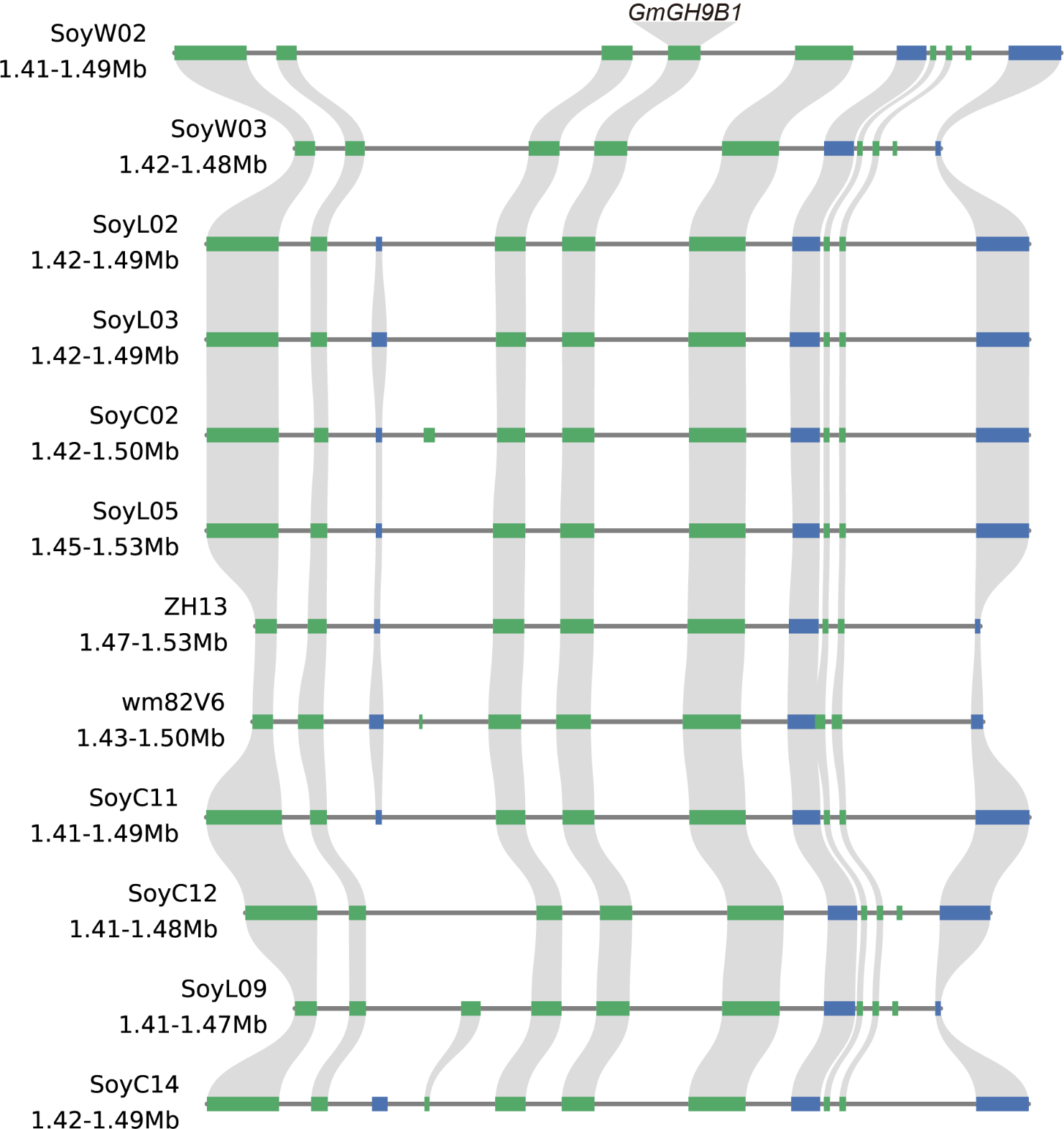


**Figure S4. Microsynteny analysis of the *GmGH9B1* within soybean species.** Green and blue represent genes on the negative and positive strands of chromosomes, respectively.


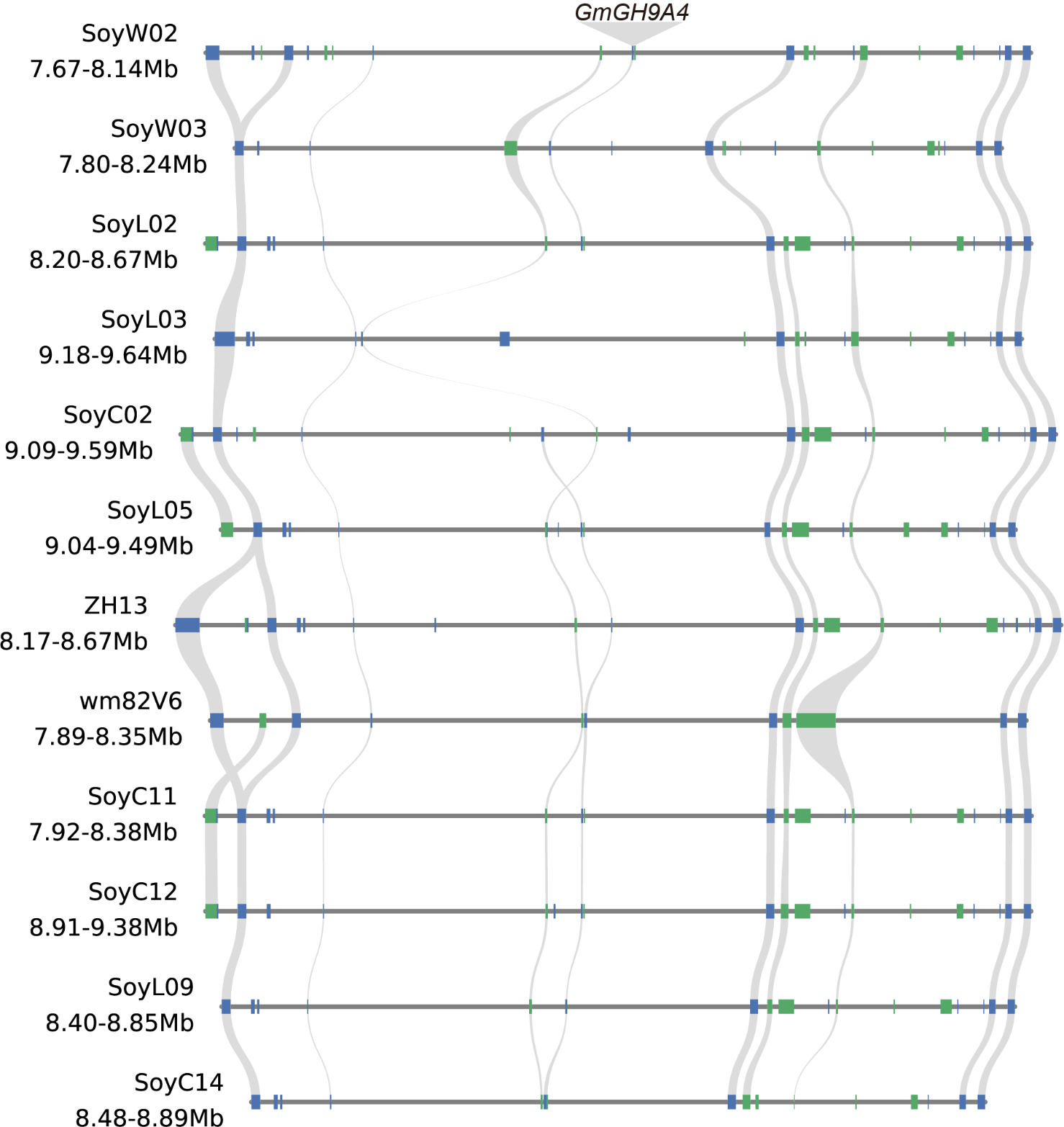


**Figure S5. Microsynteny analysis of the *GmGH9A4* within soybean species.** Green and blue represent genes on the negative and positive strands of chromosomes, respectively.


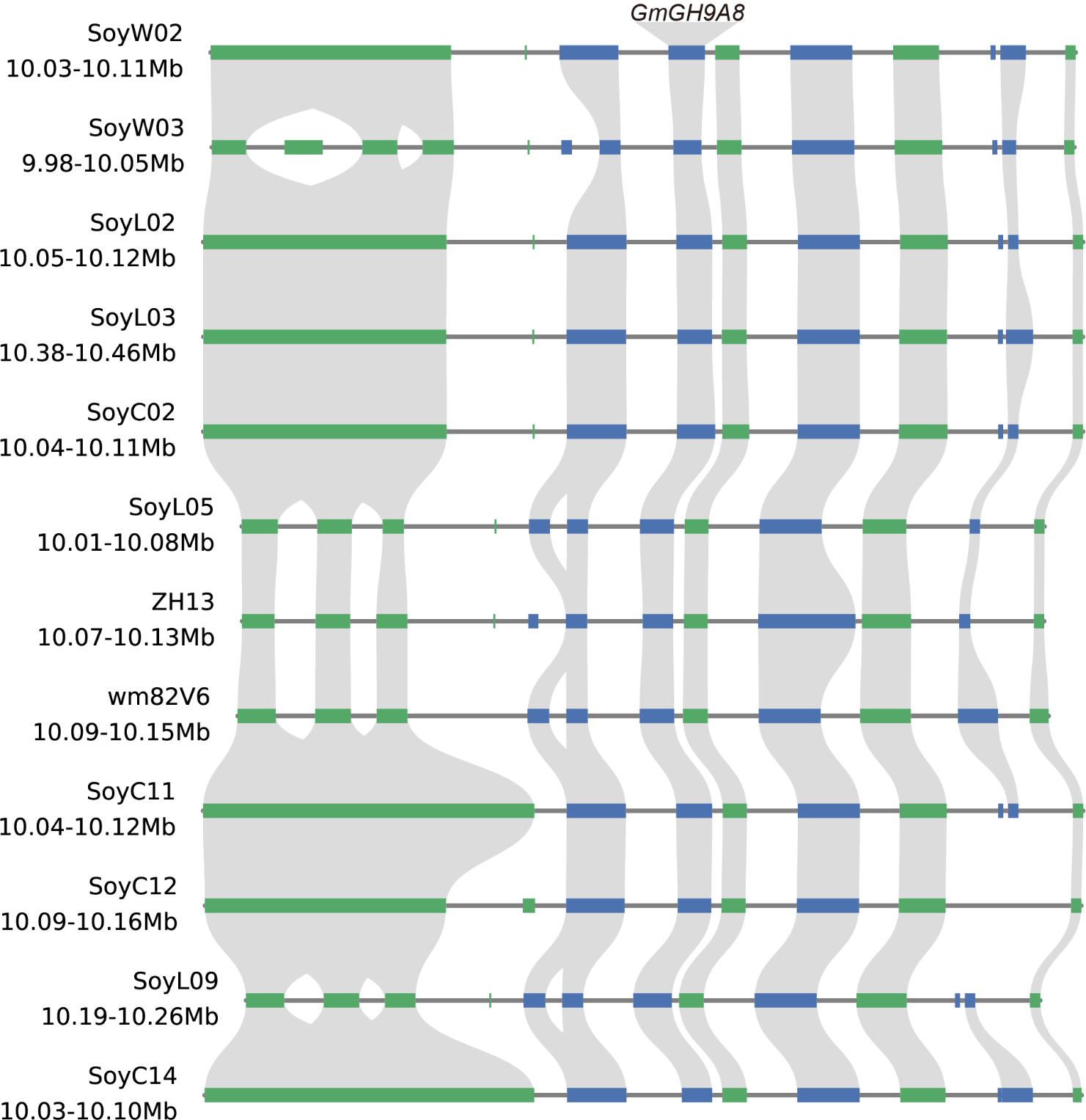


**Figure S6. Microsynteny analysis of the *GmGH9A8* within soybean species.** Green and blue represent genes on the negative and positive strands of chromosomes, respectively.


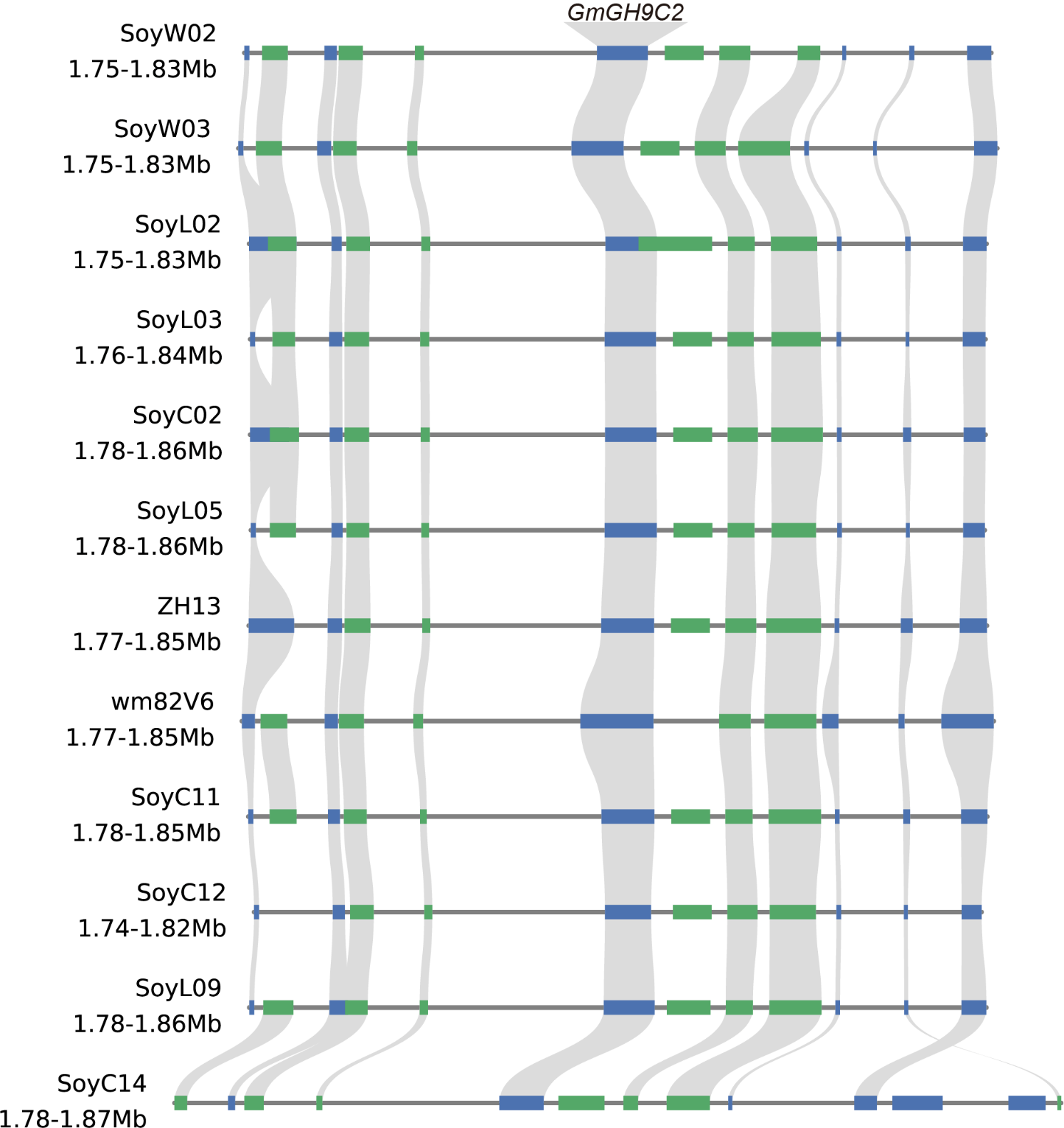


**Figure S7. Microsynteny analysis of the *GmGH9C2* within soybean species.** Green and blue represent genes on the negative and positive strands of chromosomes, respectively.


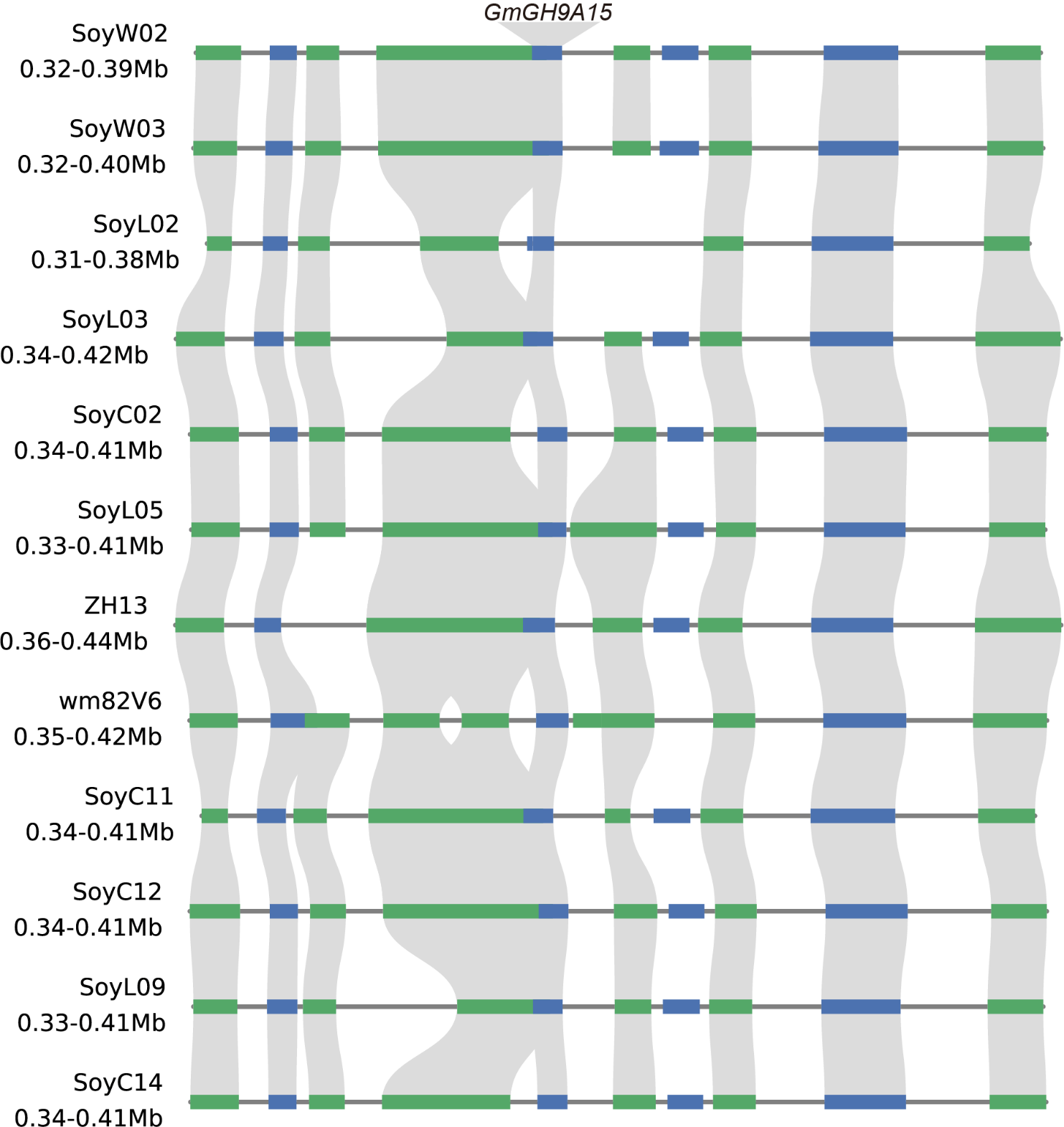


**Figure S8. Microsynteny analysis of the *GmGH9A15* within soybean species.** Green and blue represent genes on the negative and positive strands of chromosomes, respectively.


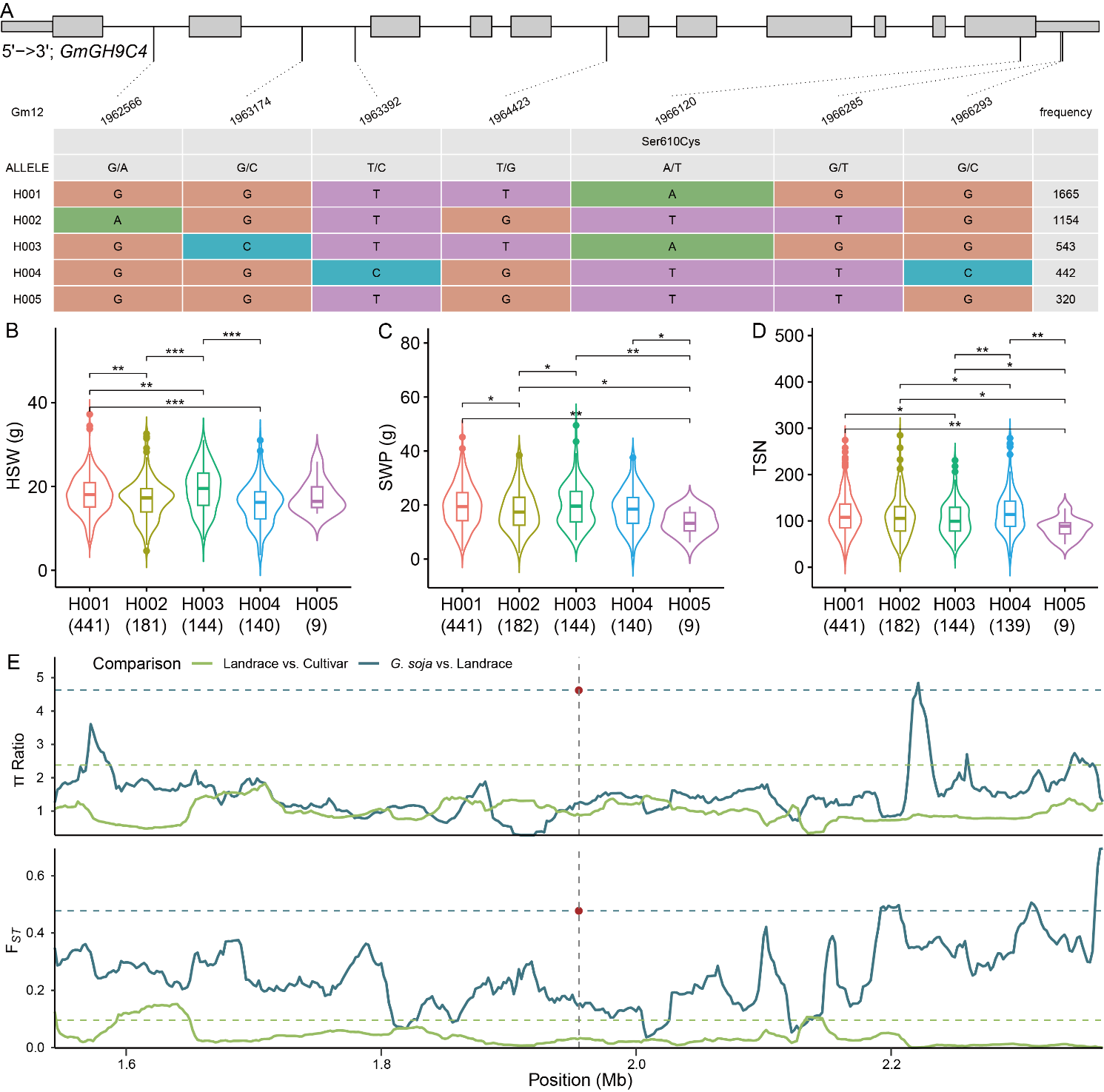


**Figure S9. Analysis of genetic characteristics of *GmGH9C4*.** (A) Haplotype of *GmGH9C4*. The wide and narrow gray boxes represent the exon and UTR regions, respectively. The gray line represents the intron. (B)-(D) The relationship between haplotypes with 100-seed weight (HSW), seed weight per plant (SWP) and total seed number (TSN). (E) The π ratio and *F*st values of flanking region at *GmGH9C4* in *G. soja*, landraces, and cultivars soybeans. The horizontal dashed lines indicate the genome-wide thresholds of *G. soja* vs landraces, and landraces vs cultivars (top 10%). *GmGH9C4* is labeled by brown dot (Gm12: 1964192). *, **, and *** indicate significant differences at *p* < 0.05, *p* < 0.01, and *p* < 0.001, respectively.
